# Catabolite repression control protein antagonist, a novel player in *Pseudomonas aeruginosa* carbon catabolite repression control

**DOI:** 10.3389/fmicb.2023.1195558

**Published:** 2023-05-12

**Authors:** Elisabeth Sonnleitner, Flavia Bassani, Anastasia Cianciulli Sesso, Paul Brear, Branislav Lilic, Lovro Davidovski, Armin Resch, Ben F. Luisi, Isabella Moll, Udo Bläsi

**Affiliations:** ^1^Department of Microbiology, Immunobiology and Genetics, Max Perutz Labs, Center of Molecular Biology, Vienna Biocenter, University of Vienna, Vienna, Austria; ^2^Vienna BioCenter PhD Program, a doctoral School of the University of Vienna and Medical University of Vienna, Max Perutz Labs, Center of Molecular Biology, Vienna Biocenter, University of Vienna, Vienna, Austria; ^3^Department of Biochemistry, University of Cambridge, Cambridge, United Kingdom

**Keywords:** carbon catabolite repression, carbon catabolite repression control protein, Hfq, *Pseudomonas*, post-transcriptional control

## Abstract

In the opportunistic human pathogen *Pseudomonas aeruginosa* (*Pae*), *c*arbon *c*atabolite *r*epression (CCR) orchestrates the hierarchical utilization of N and C sources, and impacts virulence, antibiotic resistance and biofilm development. During CCR, the RNA chaperone Hfq and the *c*atabolite *r*epression *c*ontrol protein Crc form assemblies on target mRNAs that impede translation of proteins involved in uptake and catabolism of less preferred C sources. After exhaustion of the preferred C-source, translational repression of target genes is relieved by the regulatory RNA CrcZ, which binds to and acts as a decoy for Hfq. Here, we asked whether Crc action can be modulated to relieve CCR after exhaustion of a preferred carbon source. As Crc does not bind to RNA *per se*, we endeavored to identify an interacting protein. *In vivo* co-purification studies, co-immunoprecipitation and biophysical assays revealed that Crc binds to *Pae* strain O1 protein PA1677. Our structural studies support bioinformatics analyzes showing that PA1677 belongs to the isochorismatase-like superfamily. Ectopic expression of PA*1677* resulted in de-repression of Hfq/Crc controlled target genes, while in the absence of the protein, an extended lag phase is observed during diauxic growth on a preferred and a non-preferred carbon source. This observations indicate that PA1677 acts as an antagonist of Crc that favors synthesis of proteins required to metabolize non-preferred carbon sources. We present a working model wherein PA1677 diminishes the formation of productive Hfq/Crc repressive complexes on target mRNAs by titrating Crc. Accordingly, we propose the name CrcA (*c*atabolite *r*epression *c*ontrol protein *a*ntagonist) for PA1677.

## Introduction

*Pseudomonas aeruginosa (Pae)* can utilize numerous carbon sources, enabling it to flourish in diverse environmental niches, such as soil, marine habitats as well as on/in different organisms. In humans, *Pae* is an opportunistic pathogen that can cause acute and chronic infections of immunocompromised individuals (Gellatly and Hancock, 2013).

In Bacteria, the uptake and utilization of carbon compounds is controlled in a hierarchical manner by a mechanism known as *c*arbon *c*atabolite *r*epression (CCR). In short, CCR prevents the utilization of less preferred carbon sources until the preferred one is consumed. In *E. coli* CCR prevents the expression of catabolic genes, the transcription of which requires the transcriptional activator CRP (*c*yclic AMP *r*eceptor *p*rotein) in conjunction with cAMP, whereas in *B. subtilis* CCR is mediated by the transcriptional repressor CcpA (*c*atabolite *c*ontrol *p*rotein *A*). In both organisms CCR is regulated at the transcriptional level by a signal transduction cascade inherent to the phosphoenolpyruvate-carbohydrate phosphotransferase system (Görke and Stülke, 2008).

In most studied *Pseudomonas* spp. the presence of organic acids (e.g., succinate) results in CCR, which leads to repression of catabolic genes required for the consumption of other C/N sources. In *Pae*, CCR is regulated at the post-transcriptional level (Rojo, 2010; Sonnleitner and Bläsi, 2014) by the global regulator Hfq in conjunction with the *c*atabolite *r*epression *c*ontrol protein Crc (Sonnleitner and Bläsi, 2014; Ferrara et al., 2015; Moreno et al., 2015; Sonnleitner et al., 2018). Hfq was shown to bind directly to mRNAs that contain (AAN)_n_ or (ARN)_n_ repeats, in which A is an adenine, R is a purine (A/G) and N is any nucleotide. These motifs can engage the tripartite binding pockets on the distal face of Hfq (Link et al., 2009) to block translation during CCR (Sonnleitner and Bläsi, 2014). Hereby, Crc acts as a co-repressor by stabilizing the Hfq-RNA complex (Sonnleitner et al., 2018; Malecka et al., 2021). In fact, cryo-EM studies showed that a segment encompassing the ribosome binding site of the model *amiE* mRNA is sandwiched between Hfq and Crc, rationalizing the auxiliary function of Crc in Hfq-mediated translational repression by obstructing initiating ribosomes (Pei et al., 2019). Moreover, more recent cryo-EM studies on several target mRNAs revealed that Hfq/Crc assemblies have mRNA-specific quaternary architectures, resulting from the combination of multivalent protein–protein interfaces and recognition of patterns in the RNA sequence. The structural polymorphism of these ribonucleoprotein assemblies can most likely explain the repression of many different target mRNAs (Dendooven et al., 2023).

The response to different C-sources was shown to be mediated through different levels of the regulatory RNA CrcZ (Sonnleitner et al., 2009; Valentini et al., 2014). Owing to several Hfq binding motifs, CrcZ can titrate Hfq and/or form Hfq/Crc/CrcZ complexes after alleviation of CCR (Sonnleitner and Bläsi, 2014; Moreno et al., 2015; Sonnleitner et al., 2018). This in turn leads to the translation of different metabolic genes and to the utilization of non-or less preferred C/N sources. In addition, Hfq/Crc control virulence gene expression (Sonnleitner et al., 2003, 2006), quorum sensing (Kimyon et al., 2016; Yang and Lan, 2016), biofilm formation (O’Toole et al., 2000; Pusic et al., 2016) as well as antibiotic susceptibility (Linares et al., 2010; Pusic et al., 2018; Sonnleitner et al., 2020).

Several studies have provided insights into the mechanism of translational repression by Hfq/Crc (Moreno et al., 2015; Sonnleitner et al., 2018; Pei et al., 2019; Dendooven et al., 2023). More recently, single-molecule fluorescence assays revealed that the presence of Crc does not change the Hfq-target RNA interaction lifetimes, whereas it changes the equilibrium toward more stable repressive complexes. This observation is in accord with Cryo-EM analyzes, which showed an increased compactness of the repressive Hfq/Crc/RNA assemblies (Malecka et al., 2021). In contrast, nothing is known about the fate of stable Hfq/Crc/RNA complexes or the avoidance of continued formation of stable Hfq/Crc/CrcZ assemblies after relief of CCR, which might be important in light of the requirement of free Hfq for riboregulation by sRNAs (Sonnleitner et al., 2017; Pusic et al., 2021), its impact on biofilm formation (Pusic et al., 2016) and antibiotic susceptibility (Pusic et al., 2018; Sonnleitner et al., 2020).

In this study, we asked whether a protein interacting with Crc might affect Hfq/Crc/RNA complex formation upon relief of CCR. We reasoned that such a Crc antagonist could reduce the continued formation of stable Hfq/Crc/RNA complexes and assist in increasing the pool of free Hfq under these conditions. Using affinity purification in combination with mass spectrometry we identified proteins that interact with Crc, with PA1677 as one of them. Our crystallographic results support the notion that PA1677 belongs to the isochorismatase-like superfamily. We observed that PA1677 interfered with Hfq/Crc-mediated translational repression of the model *amiE* mRNA, encoding an aliphatic amidase (Sonnleitner and Bläsi, 2014), and PA*2338*, the first gene of an operon involved in the uptake and utilization of mannitol. Immunological as well as biophysical studies supported a physical interaction of PA1677 with Crc. Moreover, we provide evidence that PA1677 accumulates during the lag phase of diauxic growth and decreases its duration. As PA1677 apparently counteracts the function of Crc, we termed the protein CrcA (*c*atabolite *r*epression *c*ontrol protein *a*ntagonist).

## Materials and methods

### Bacterial strains, plasmids and growth conditions

Detailed information on plasmids and strains used in this study is provided in Supplementary Text S1; Supplementary Table S1. Unless indicated otherwise, the cultures were grown in Basal-Salt medium (BSM) (30.8 mM K_2_HPO_4_, 19.3 mM KH_2_PO_4_, 15 mM (NH_4_)SO_4_, 1 mM MgCl_2_ and 2 μM FeSO_4_) supplemented with the indicated carbon sources or in BSM complex medium (Sonnleitner et al., 2018; BSM medium containing 40 mM succinate, 5 mM each of acetate, glucose, mannitol, acetamide, histidine, tryptophan, phenylalanine, leucine, isoleucine, glutamate, arginine, valine, lysine, 0.25 mM anthranilate and 0.25% v/v glycerol). If required, strain *Pae* strain O1 (PAO1) was grown in the presence of 250 μg/ml carbenicillin, 100 μg/ml tetracycline or 50 μg/ml gentamicin. If not indicated otherwise, genes controlled by the P*tac*-promoter in the plasmid pMMB67HE-derivatives were induced in the presence of 1 mM (final concentration) of isopropyl β-D-1-thiogalactopyranoside (IPTG).

### Identification of Strep-Crc interacting proteins by LC–MS/MS

PAO1Δ*crc* was transformed with plasmid pMMB-Strep-*crc* and grown at 37°C in BSM complex medium. The cells were harvested at an OD_600_ of 1.5 and the cell pellet was resuspended in lysis buffer (50 mM Tris–HCl pH 8.0, 150 mM NaCl, 1 mM PMSF, 1 mM β-mercaptoethanol) and lysed by sonication. A PAO1Δ*crc* lysate was obtained under the same conditions to serve as a mock control. Strep-Crc interacting proteins were co-purified by affinity chromatography following the standard protocol for the Strep-Tactin® resin (IBA). The identity of all proteins present in the mock control and those co-purifying with Strep-Crc were analyzed by mass spectrometry at the Vienna BioCenter Core Facility^1^ as described in the Supplementary Text S1.

### RNA_seq_ library construction and sequence analysis

Total RNA was prepared from two biological replicates of strains PAO1ΔPA*1677*(pMMB67HE) and PAO1ΔPA*1677*(pMMB-Strep-*1677*) after growth in BSM complex medium to an OD_600_ of 1.5. 10 ml samples were withdrawn and total RNA was extracted using the hot phenol method (Leoni et al., 1996), contaminating DNA was removed by TURBO™ DNase (Thermo Fisher Scientific) treatment followed by phenol-chloroform (pH 5.5) extraction and ethanol precipitation. Ribosomal RNA (rRNA) depletion, library preparation, sequencing and initial data processing were performed by Lexogen, Austria. In brief, rRNAs were removed with the RiboCop rRNA Depletion Kit for Gram-negative Bacteria. The libraries were constructed using Lexogen’s CORALL™ Total RNA-Seq Kit. The samples were sequenced with an Illumina NextSeq 2000 platform using 100 nt single end read length. Sequencing quality control of the raw reads was assessed using FastqQC^2^ software and adaptor sequences were removed with cutadapt (Martin, 2011). Alignment to the PAO1 reference genome (NCBI accession number NC_002516.2) and read counting were performed using STAR (Dobin et al., 2013). The DESeq2 R package (Love et al., 2014) was used to perform differential gene expression analysis of PAO1ΔPA*1677* harboring the PA*1677* over-expressing plasmid versus strain PAO1ΔPA*1677* bearing the parental plasmid. Gene annotations were acquired from the *Pseudomonas* Genome Database (Winsor et al., 2016). All RNAs with a fold-change greater than 2 and a multiple testing adjusted value of p below 0.05 were considered to be differentially abundant. The raw sequencing data were deposited in the European nucleotide archive (ENA) as a study under the accession number PRJEB60904.

### β-galactosidase assays

The β-galactosidase activities were determined as described (Miller, 1972). The strains indicated in the corresponding Figure legends were grown in BSM medium supplemented with 40 mM succinate and either 40 mM acetamide or 5 mM mannitol to induce *amiE* and PA*2338* transcription, respectively. At an OD_600_ of 2.0, the cells were harvested and permeabilized with 5% toluene. The β-galactosidase activities were derived from three independent experiments and presented as mean with error bars corresponding to standard deviations.

### Purification of proteins

The synthesis of the proteins His-Crc, Strep-PA1677 (Strep-CrcA) and His-PA1677 (His-CrcA) in strains PAO1 (pMMB-His-3C-Crc), PAO1Δ*crc*(pMMB-Strep-*1677*) and PAO1Δ*crc*(pMMB-His-3C-*1677*), respectively, was induced by addition of IPTG to a final concentration of 1 mM for 3 h after growth at 37°C in LB medium. The cells were harvested by centrifugation, the pellets were resuspended in lysis buffer (50 mM Tris–HCl pH 8.0, 300 mM NaCl, 10 mM imidazole, 1 mM β-mercaptoethanol, 20 μg/ml lysozyme and 20 μg/ml RNaseA), and lysis was accomplished using a single cycle in a cell disruptor (Constant Systems Ltd., United Kingdom), with the pressure set at 1.9 kPa. After lysis, 20 μg/ml DNaseI and 0.1 mM phenylmethylsulphonyl fluoride (PMSF) were added. His-Crc and His-CrcA were further purified by Ni-affinity chromatography following the protocol provided by the manufacturer (Qiagen, Germany).

The His-tag of Crc was removed by GST-HRV14-3C “PreScission” cleavage followed by Ni-affinity chromatography and Superdex-75 size-exclusion chromatography in the presence of 50 mM HEPES pH 8.0, 150 mM NaCl and 1 mM β-mercaptoethanol as described (Milojevic et al., 2013). His-CrcA was dialyzed against 50 mM Tris–HCl pH 8.0, 25 mM NaCl, 1 mM β-mercaptoethanol and loaded on a Pierce™ Strong Anion Exchange Mini Spin Column (Thermo Fisher Scientific, United States). His-CrcA was eluted with 50 mM Tris–HCl pH 8.0, 80 mM NaCl, 1 mM β-mercaptoethanol. The His-tag of His-CrcA was removed by GST-HRV14-3C “PreScission” cleavage followed by further purification using Ni-affinity chromatography and Superdex-75 size-exclusion chromatography in the presence of 50 mM Tris pH 8.0, 150 mM NaCl and 1 mM β-mercaptoethanol. The resulting CrcA protein was used for grating-coupled interferometry.

Strep-CrcA was purified by affinity chromatography following a standard protocol of the Strep-Tactin®XT Superflow® high capacity resin (IBA GmbH, Germany). The Strep-CrcA protein fraction was further purified by Superdex-75 size-exclusion chromatography in the presence of 50 mM Tris–HCl pH 8.0, 150 mM NaCl, 1 mM β-mercaptoethanol.

The PAO1 Hfq protein was produced in the *hfq* deficient *E. coli* strain JW4130F´ harboring the plasmid pKEHfq_Pae_. The purification was performed as described by Beich-Frandsen et al. (2011).

### Structural studies

Strep-CrcA and Crc were mixed at 42.6 μM each, concentrated with a 10 kDa MWCO vivaspin 2 centrifugal concentrator to 335 μM, and used in co-crystallization screens. A second set of crystallization trials were performed with a mixture of Strep-CrcA and Crc at 335 μM and 169 μM, respectively, corresponding to roughly a 2:1 ratio. Crystals appeared under different conditions and in several different space groups (Supplementary Table S2). The data were collected at the Diamond Light source station I24. Molecular replacement solutions using PHASER (McCoy et al., 2007) with models for Crc and CrcA found only CrcA. The models were refined with PHENIX (Liebschner et al., 2019) and optimized with COOT (Emsley et al., 2010). The structure was refined from the solution of the C222_1_ crystal (PDB accession number 8CIB) and the corresponding protomer was used for molecular replacement solutions of the other space groups (Supplementary Table S2). The refinement parameters of the C222_1_ crystal structure is shown in Supplementary Table S3.

### *In vitro* co-immunoprecipitation studies

For the *in vitro* Co-IP studies, 50 pmol of Crc protein and/or 50 pmol of Strep-CrcA were incubated for 15 min at 37°C in 200 μl ES-buffer (10 mM Tris pH 8.0, 10 mM KCl, 40 mM NaCl and 1 mM MgCl_2_) in the presence of 0.05% Triton X-100. Then, 10 μl of rabbit anti-Crc antibodies (Pineda, Germany) were added, and the incubation was continued for 2 h on ice. Then, 10 μl Dynabeads® Protein G beads (Novex) were added, and the incubation was continued overnight at 4°C on a rotating wheel. The beads were washed four times with ES-buffer and finally collected in 50 μl of SDS loading dye. 5 μl were used for Western-blotting.

### Western-blot analyzes

The proteins were separated on 12.5% SDS polyacrylamide gels and electro-transferred at 25 V for 30 min onto a 0.2 μm nitrocellulose membrane (GE Healthcare, Germany) using transfer buffer (50 mM Tris base, 40 mM glycine, 0.04% (w/v) SDS, 20% (v/v) methanol, pH 8.3), and then probed with rabbit anti-PA1677 (Pineda, Germany), anti-Crc (Pineda, Germany), anti-Strep-tag II (PromoCell, Germany) or anti-S1 (Pineda, Germany) antibodies, respectively. Anti-rabbit IgG coupled to horseradish peroxidase (Cell Signaling Technology, USA) was used as a secondary antibody and the blot was developed using a chemiluminescent reagent (SuperSignal West Pico PLUS, Thermo Scientific, United States). The BioRad ChemiDoc™ MP Imaging system was used for signal detection.

### Grating-coupled interferometry

The binding kinetics of CrcA and Crc were determined at the Vienna BioCenter Core Facility (see text footnote link 1) using the Creoptix® WAVEdelta system (Creoptix AG, Switzerland). The biosensor system for label-free detection of binding kinetics in real-time is based on grating-interferometry. CrcA and Crc were immobilized on the surface of separate channels of the WAVEchip 4PCP (Creoptix AG) by amine coupling using the standard amine labeling kit from Xantec, Germany. Briefly, the polycarboxylate hydrogen layer of the 4PCP sensor chip surface was activated by 1-Ethyl-3-(3-diethylaminopropyl)-carbodiimide-hydrochloride (EDC) and N-hydroxysuccinimide (NHS). Then, 30 μg/ml solutions of CrcA in sodium acetate (10 mM, pH 5) and Crc in ES-buffer (10 mM Tris/HCl pH 8, 10 mM KCl, 40 mM NaCl and 1 mM MgCl_2_), respectively, were applied to the chip with a flow rate of 10 μl/min. The different buffer for Crc was required because the protein was unstable at low pH. The final surface densities for the channel with CrcA immobilization were 9.5 ng/mm^2^ and with Crc immobilization 15 pg/mm^2^. No protein-ligand was applied in the reference channel. After immobilization the sensor chip surface was blocked with 1 M ethanolamine/HCl pH 8.5. As running buffer filtered (pore size: 0.22 μm) and degassed ES-buffer was used. For the kinetic assay, the Crc and CrcA proteins were serially diluted in ES-buffer as indicated in the corresponding Figure legends. As a control Hfq protein was used alone (negative control) or mixed in a 1:1 ratio with *amiE*_6ARN_ RNA-oligonucleotide (5′-AAA AAU AAC AAC AAG AGG-3′; purchased from Sigma-Aldrich, United States), resulting in formation of Hfq/Crc/*amiE*_6ARN_ RNA assemblies that served as a positive control for Crc binding (Sonnleitner et al., 2018). The experiments were carried out at flow rates of 100 μl/ml for the interaction studies with Crc and CrcA (due to the fast on and off rates) and 30 μl/ml for the controls at 25°C. The analysis was performed with the analysis tool of the Creoptix WAVEcontrol program 4.1.0. The experiments were performed in duplicate.

## Results

### Identification of Crc interacting proteins

With the aim to isolate a protein factor that interacts with Crc, Strep-tagged Crc was captured by using the Strep-tag technology after growth of strain PAO1Δ*crc* harboring plasmid pMMB-Strep-*crc* in BSM complex medium. Unspecific binding to the affinity matrix was controlled by a mock purification under the same conditions using cell lysates of strain PAO1Δ*crc* harboring the parental plasmid pMMB67HE. After elution from the Strep-Tactin® resin, the proteins co-purifying with Strep-Crc (Supplementary Figure S1) were identified by mass spectrometry. Protein identifications were accepted with a probability and sequence coverage greater than 99 and 50%, respectively, and with a minimum of 10 unique peptides. In addition, positive candidates were only considered when ≤1 peptide of the respective protein was present in the mock control. These constraints revealed-with the exception of Strep-Crc-only 3 proteins, one of which was Hfq, known to form complexes on target mRNAs together with Crc (Moreno et al., 2015; Sonnleitner et al., 2018; Pei et al., 2019). In addition, this study identified PA1677, a putative isochorismatase family hydrolase, and PA3919, a PhoH like protein (Supplementary Table S4). According to the criteria above mentioned for the mass spectrometry analysis, PA1677 emerged as a prime candidate for a Crc interacting protein.

### PA1677 synthesis leads to de-repression of genes controlled by CCR

Serving as a co-repressor, Crc contributes to translational repression of Hfq (Sonnleitner and Bläsi, 2014; Pei et al., 2019; Malecka et al., 2021). Translational repression is frequently accompanied with reduced stability and reduced abundance of target mRNAs (Sonnleitner et al., 2018). Therefore, we performed an RNA-seq based transcriptome analysis and asked whether ectopic expression of PA*1677* might alter the abundance of Hfq/Crc controlled genes. For this purpose, strain PAO1ΔPA*1677*, carrying an in-frame deletion of PA*1677* was constructed and transformed with plasmid pMMB-Strep-*1677* or the parental vector pMMB67HE. The strains were grown to an OD_600_ of 1.5 in BSM-complex medium (Sonnleitner et al., 2018). As succinate is the preferred carbon source of PAO1 it was included in the medium to establish CCR. The other C and N sources were added to induce transcription of the respective CCR-controlled genes (Sonnleitner et al., 2018). The synthesis of Strep-PA1677 was induced in the presence of 1 mM IPTG, and its synthesis was demonstrated by Western-blot analysis using anti-PA1677 specific antibodies (Supplementary Figure S2). According to the criteria mentioned above for the RNA-seq analysis, 116 transcripts were differentially abundant in the presence of Strep-PA1677 when compared to its absence (Supplementary Table S5). Among these transcripts, 112 were up-regulated and 4 were down-regulated. These transcripts were further compared with RNA-seq analyzes performed previously with the strains PAO1Δ*hfq* and PAO1Δ*crc* under the same conditions (Sonnleitner et al., 2018). We considered only transcripts that were up-regulated in the absence of either, *hfq* and/or *crc*, to be under direct CCR control. Twenty-six overlapping transcripts were identified (Table 1). Among them, the well-studied CCR target *amiE* as well as the accompanying operon genes were recognized (Sonnleitner et al., 2009, 2018). In addition, the *bkdA1*, *bkdA2*, *bkdB* and *lpdV* operon required for the utilization of branched chain amino-acids was up-regulated upon ectopic expression of *strep-*PA*1677* (Table 1). Furthermore, the genes required for the uptake and/or assimilation of the less preferred C-sources mannitol (PA*2338-*PA*2343*) and glucose (PA*3186-*PA*3189*), which are known to be under CCR control (Wolff et al., 1991), were likewise up-regulated (Table 1; Sonnleitner et al., 2018). Taken together, the RNA-seq analysis supported the notion that PA1677 might interfere with the formation of Hfq/Crc repressive complexes on known target mRNAs.

**Table 1:** Transcripts with increased abundance in PAO1*Δ*PA*1677*(pMMB-Strep-*1677*) when compared with PAO1*Δ*PA*1677*(pMMB67HE).

PA-number	Fold change	padj	Gene name	Description
PA2247	2.66	3.14E-08	*bkdA1*	2-oxoisovalerate dehydrogenase (alpha subunit)
PA2248	2.64	3.32E-07	*bkdA2*	2-oxoisovalerate dehydrogenase (beta subunit)
PA2249	2.51	9.12E-07	*bkdB*	Branched-chain alpha-keto acid dehydrogenase (lipoamide component)
PA2250	2.78	4.13E-06	*lpdV*	Lipoamide dehydrogenase-Val
PA2338	4.24	9.03E-16	PA*2338*	Probable binding protein component of ABC maltose/mannitol transporter
PA2339	4.97	3.00E-07	PA*2339*	Probable binding-protein-dependent maltose/mannitol transport protein
PA2340	3.98	1.94E-04	PA*2340*	Probable binding-protein-dependent maltose/mannitol transport protein
PA2341	5.09	6.80E-13	PA*2341*	Probable ATP-binding component of ABC maltose/mannitol transporter
PA2342	4.43	3.10E-09	*mtlD*	Mannitol dehydrogenase
PA2343	5.74	1.23E-04	*mtlY*	xylulose kinase
PA2389	3.69	7.07E-07	*pvdR*	Siderophore efflux pump protein PvdR
PA2553	2.11	1.38E-02	PA*2553*	Probable acyl-CoA thiolase
PA2554	2.37	1.38E-02	PA*2554*	Probable short-chain dehydrogenase
PA3186	4.29	2.97E-07	*oprB*	Glucose/carbohydrate outer membrane porin OprB precursor
PA3187	9.26	7.80E-18	PA*3187*	Probable ATP-binding component of ABC transporter
PA3188	14.32	2.74E-8	PA*3188*	Probable permease of ABC sugar transporter
PA3189	12.67	4.63E-12	PA*3189*	Probable permease of ABC sugar transporter
PA3190	41.81	2.51E-71	PA*3190*	Probable binding protein component of ABC sugar transporter
PA3362	6.21	3.37E-18	*amiS*	Hypothetical protein
PA3363	10.16	2.76E-38	*amiR*	Aliphatic amidase regulator
PA3364	7.74	2.61E-32	*amiC*	Aliphatic amidase expression-regulating protein
PA3365	10.84	1.42E-48	*amiB*	Probable chaperone
PA3366	9.76	4.52E-43	*amiE*	Aliphatic amidase
PA4022	3.00	2.41E-04	*hdhA*	Hydrazone dehydrogenase, HdhA
PA5168	2.41	1.29E-02	*dctQ*	C(4)-dicarboxylate transport system protein DctQ
PA5169	3.27	3.02E-04	*dctM*	C(4)-dicarboxylate transport system protein DctM

These transcripts are also up-regulated under the same growth conditions in PAO1Δ*hfq* and PAO1Δ*crc*, respectively, when compared with the wild-type strain PAO1 (Sonnleitner et al., 2018). Single genes or genes situated in operons are highlighted by alternate colour.

**Figure 1:**
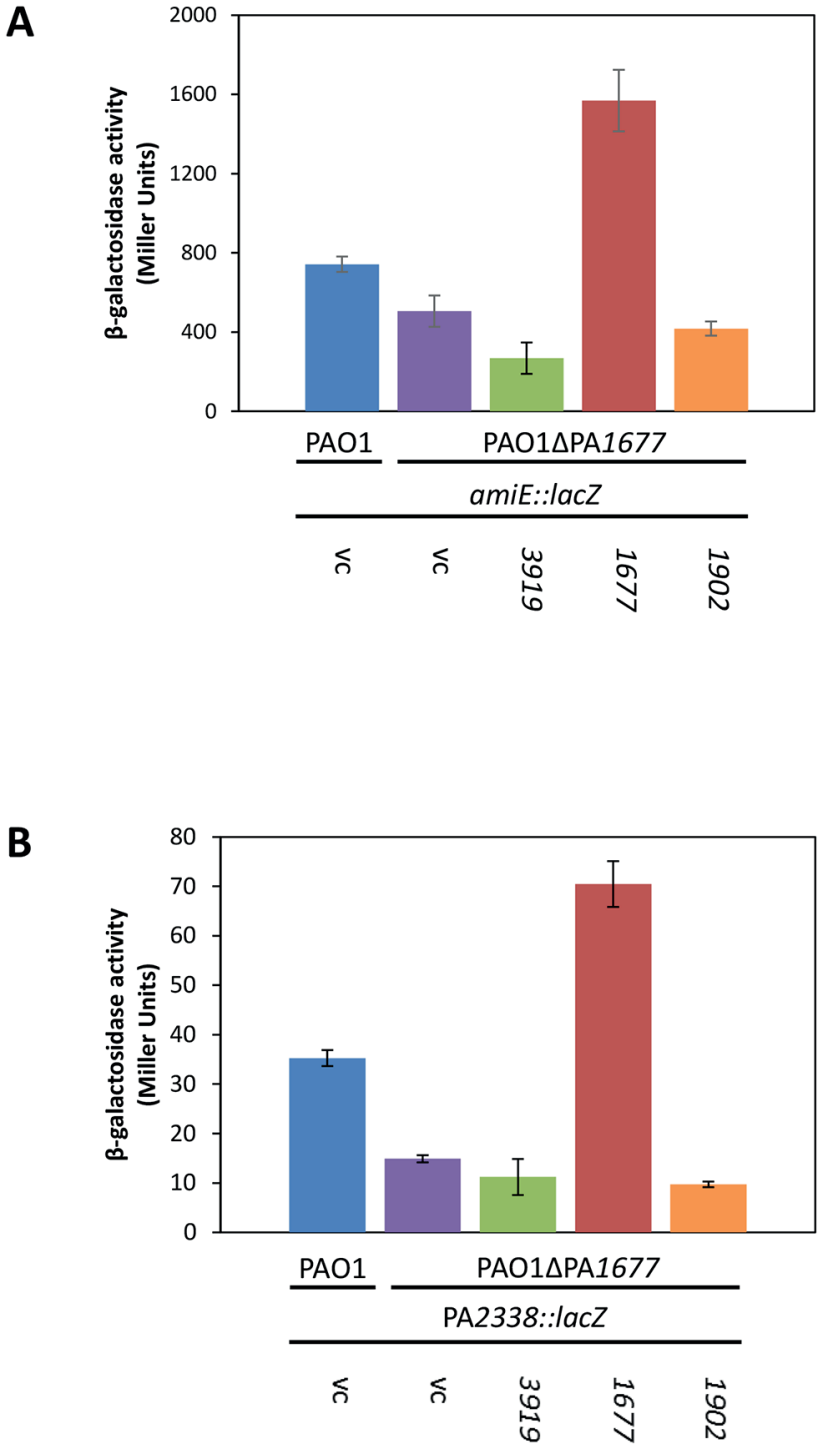
Synthesis of Strep-PA1677 results in de-repression of *amiE* and PA*2338* translation. **(A)** The strains PAO1(pMMB67HE; vc, vector control), PAO1ΔPA*1677*(pMMB67HE; vc), PAO1ΔPA*1677*(pMMB-Strep-*3919; 3919*), PAO1ΔPA*1677*(pMMB-Strep-*1677; 1677*) and PAO1ΔPA*1677*(pMMB-Strep-*1902; 1902*) carried in addition plasmid pME9655, encoding an *amiE::lacZ* translational reporter gene. **(B)** The strains PAO1(pMMB67HE; vc), PAO1ΔPA*1677*(pMMB67HE; vc), PAO1ΔPA*1677*(pMMB-Strep-*3919; 3919*), PAO1ΔPA*1677*(pMMB-Strep-*1677*; *1677*) and PAO1ΔPA*1677*(pMMB-Stre*p-1902*; *1902*) carried in addition plasmid pME6015-PA*2338*, encoding an PA*2338::lacZ* translational reporter gene. All strains were grown in BSM-succinate to an OD_600_ of 2.0. Acetamide (40 mM final concentration; **A**) and mannitol (5 mM final concentration, **B**) were added to induce transcription of *amiE::lacZ* and PA*2338::lacZ*, respectively. The bars represent the β-galactosidase values conferred by the AmiE-LacZ protein **(A)** and the PA2338-LacZ protein **(B)**, respectively. All measurements were performed in triplicate. The error bars represent standard deviations.

To assess directly whether PA1677 interferes with Hfq/Crc-mediated regulation, we made use of a translational *amiE::lacZ* reporter gene inserted into plasmid pME9655. AmiE-LacZ synthesis was previously shown to be repressed by Hfq and Crc (Sonnleitner and Bläsi, 2014), and in the above described RNA-seq analysis the *amiE* transcript was shown to be more abundant in strain PAO1ΔPA*1677*(pMMB-Strep-*1677*) when compared with strain PAO1ΔPA*1677*(pMMB67HE; Table 1). The synthesis of the AmiE-LacZ protein was first assessed in strains PAO1(pME9655, pMMB67HE) and PAO1ΔPA*1677*(pME9655, pMMB67HE). When compared with PAO1, the *amiE::lacZ* gene was slightly more repressed in strain PAO1ΔPA*1677* (Figure 1A), indicating that PA1677 might interfere with translational regulation by Hfq/Crc.

To test this hypothesis, the strain PAO1ΔPA*1677*(pME9655) was transformed with plasmid pMMB-Strep-*1,677*, harboring the *strep-*PA*1677* gene under transcriptional control of the *tac* promoter. As shown in Figure 1A, *amiE::lacZ* translation was significantly de-repressed when Strep-PA1677 was co-synthesized (Supplementary Figure 3). Using the same genetic setup, we also tested whether co-synthesis of Strep-PA3919, which was identified as another putative interactor of Crc (Supplementary Table S4), can interfere with *amiE::lacZ* translation. As PA1677 was predicted to belong to the isochorismatase family (Winsor et al., 2016), Strep-PhzD2 (Strep-PA1902), which is a known isochorismatase involved in phenanzine biosynthesis (Mavrodi et al., 2001; Parsons et al., 2003), was additionally co-produced with AmiE-LacZ (Figure 1A). However, co-synthesis of either Strep-tagged protein (Supplementary Figure S3) did not result in de-repression of the *amiE::lacZ* gene (Figure 1A), indicating the observed de-repression of *amiE::lacZ* translation can be specifically attributed to Strep-PA1677.

To verify these results, an additional Hfq/Crc target gene was employed. Gene PA*2338* is the first gene of an operon consisting of 7 genes encoding putative ABC mannitol/mannose transporter proteins (PA2338-PA2341), mannitol dehydrogenase (MtlD), xylose kinase (MtlY) and fructokinase (MtlZ) (Winsor et al., 2016). Our recent RNA-seq analyzes and metabolomics studies suggested that the operon is regulated by Hfq/Crc during CCR (Sonnleitner et al., 2018; Rozner et al., 2022). Moreover, the RNA-seq study performed herein (Table 1) suggested that PA1677 might interfere with its regulation. Translational regulation of PA*2338* by Hfq/Crc was first verified by employing a translational PA*2338::lacZ* reporter gene mounted on plasmid pME6015-PA*2338*. As anticipated, and shown in Supplementary Figure S4, the translation of PA*2338::lacZ* was de-repressed in both, PAO1Δ*crc* and PAO1Δ*hfq*, i.e., in the absence of Crc and Hfq. As shown in Figure 1B, PA*2338::lacZ* translation was repressed by ~50% in strain PAO1ΔPA*1677* when compared with strain PAO1. Furthermore, co-synthesis of Strep-PA1677 resulted in de-repression of the PA*2338::lacZ* reporter gene, whereas no de-repression of PA*2338::lacZ* was observed upon co-synthesis of Strep-PA3919 and Strep-PA1902, respectively (Figure 1B). As co-synthesis of PA3919 did not affect translation of the reporter genes, we did not further consider this protein as a possible candidate interfering with Hfq/Crc mediated repression of target mRNAs. Hence, these studies suggested that the observed de-repression of the reporter genes results from a specific interaction of PA1677 with Crc rather than from the co-synthesis of other proteins. As PA1677 apparently associates with and counteracted Crc, the protein was termed CrcA (*c*atabolite *r*epression *c*ontrol protein *a*ntagonist). This designation is henceforth used throughout the text.

### Structure of CrcA

**Figure 2:**
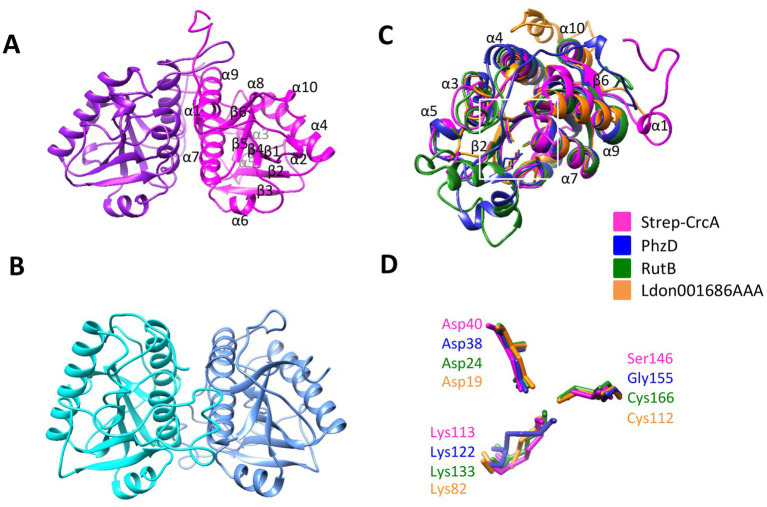
X-ray structure of Strep-CrcA in comparison with other isochorismatase-like proteins. **(A)** Crystal structure of the Strep-CrcA dimer (PDB number 8CIB). The protomers are shown in magenta and purple, respectively. **(B)** Crystal structure of the PhzD-dimer from *Pae* (PDB number 1NF9; Parsons et al., 2003). **(C,D)** Superimposition of the ribbon diagrams **(C)** and the (predicted) active site **(D)** of Strep-CrcA, PhzD, RutB (PDB number 8BLM) and the isochorismatase-like protein from *Leishmania donovani* (Ldon001686AAA; PDB number 1X9G). The area of the active site is highlighted in **(C)** by a white square.

In an attempt to shed light on the interaction of CrcA and Crc, co-crystallization trials were performed with Crc and Strep-CrcA. Diffracting crystals were obtained under several conditions and high resolution diffraction data sets were collected (Supplementary Table S2). However, only dimers of Strep-CrcA with the same self-complementary interface between the protomers were detected in the crystals (Figure 2A). The crystal space group C222_1_ and the dimerization is similar to the *Pae* PhzD (Parsons et al., 2003; Figure 2B). Superimposition of Strep-CrcA with *Pae* PhzD, *E. coli* RutB (Busch et al., 2023) and protein Ldon001686AAA of *Leishmania donovani* (Caruthers et al., 2005) revealed a high structural homology for the six β-sheets and 5 α-helices (α4, α6, α7, α9, and α10; Figure 2C; Supplementary Figure S5). However, differences were also observed between α-helix 5 and β-strand 2 (according to the consecutive nomenclature of CrcA). CrcA and Ldon001686AAA lack a sequence that is present in PhzD and RutB (Figure 2C; Supplementary Figure S5). This sequence is close to the active site and was shown to be involved in substrate binding (Parsons et al., 2003; Busch et al., 2023). The Asparagine and Lysine residues in the active site of PhzD, RutB and Ldon001686AAA are conserved in CrcA but not the Cysteine residue found in RutB and Ldon001686AAA, which is replaced by a Glycine in PhzD and by a Serine in CrcA (Figure 2C). A dimer of Strep-CrcA is consistent with the findings from SEC-MALS (*s*ize-*e*xclusion *c*hromatography coupled to *m*ulti-*a*ngle *l*ight *s*cattering), which provided an experimental mass of 41.60 kDa, which is close to the expected mass of 44.72 kDa (predicted mass of the monomer: 22.36 kDa; Supplementary Figure S6). These structural studies strongly support the notion that CrcA belongs to the isochorismatase protein family.

### *C*atabolite *r*epression *c*ontrol protein *a*ntagonist interacts with Crc

**Figure 3:**
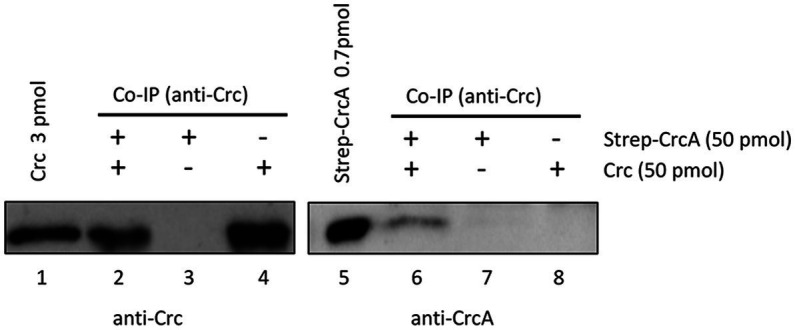
Co-Immunoprecipitation of Strep-CrcA with Crc. The Co-IP experiments were performed with anti-Crc specific antibodies, Strep-CrcA, Crc or a mixture of Strep-CrcA and Crc as indicated, and magnetic protein G beads. Lanes 1 and 5, 3 pmol Crc and 0.7 pmol Strep-CrcA protein were loaded, respectively. Lanes 2 and 6, Co-IP with anti-Crc antibodies in the presence of Crc and Strep-CrcA. The interaction of Strep-CrcA with Crc is shown after Western-blot analysis using anti-CrcA specific antibodies (right panel, lane 6). Lanes 3–4 and 7–8, control experiments in the presence of either only Strep-CrcA (lanes 3 and 7) or Crc (lanes 4 and 8).

As the Strep-CrcA/Crc crystallization studies and the SEC-MALS did not reveal a direct interaction between both proteins, we deemed the interaction either to be very weak or too transient under these experimental conditions. In a further attempt to verify the interaction of CrcA and Crc, we next performed *in vitro* co-immunoprecipitation assays (Co-IP). Anti-Crc antibodies were added to suspensions containing purified Crc, purified Strep-CrcA or to an equimolar mixture of both, and subsequently captured with magnetic protein G-beads. The beads were eluted, and the proteins were resolved on SDS-polyacrylamide gels followed by detection of Strep-CrcA and Crc by Western-blotting using anti-CrcA and anti-Crc antibodies, respectively. As shown in Figure 3 (lane 6), Strep-CrcA was co-captured with anti-Crc antibodies in the presence of Crc protein. The absence of Strep-CrcA in the Co-IP sample, which contained only Strep-CrcA, confirmed that the anti-Crc antibody does not non-specifically recognize Strep-CrcA. Hence, this Co-IP assay with purified Strep-CrcA and Crc indicated that the proteins physically interact with each other.

**Figure 4:**
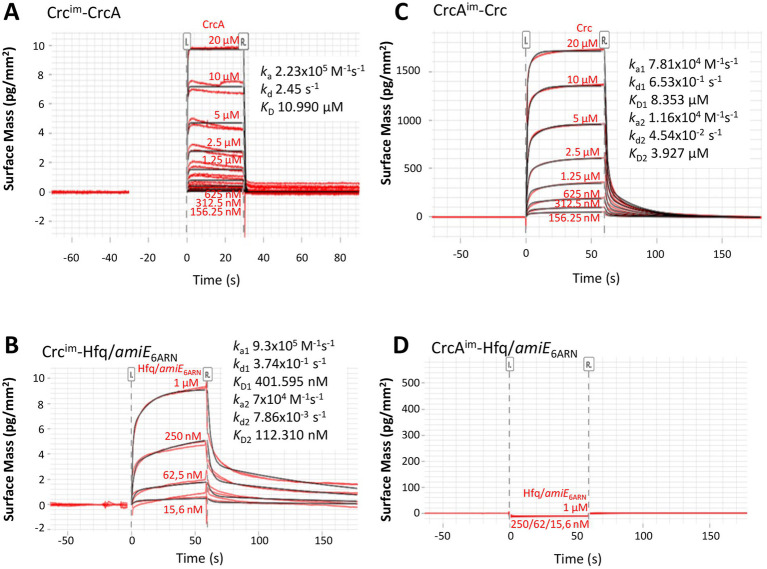
Grating coupled interferometry (GCI)-derived binding kinetics for immobilized **(A)** Crc with CrcA and **(C)** immobilized CrcA with Crc. **(B,D)** Positive and negative controls. **(B)** immobilized Crc that forms a complex with Hfq/*amiE*_6ARN_ and **(D)** immobilized CrcA that does not bind to Hfq/*amiE*_6ARN._The sensorgrams with the data in red and their respective fits in black are depicted and the corresponding kinetic parameters are shown (*k*_a_, association rate constant; *k*_d_ dissociation rate constant; *K*_D_, dissociation constant). The best fit for the kinetic analysis in **(A)** was a 1:1 kinetics and in **(B)** and **(C)** a heterogeneous ligand model.

Next, grating coupled interferometry (GCI), a surface plasmon resonance (SPR) related approach, was used to confirm the interaction between CrcA and Crc. Crc was immobilized at a low concentration (15 pg/mm^2^) to the surface of the 4-PCP-WAVE-chip (see Materials and Methods), and then serial dilutions of CrcA protein (from 20 μM to 156.25 nM) were added. The corresponding sensorgram is shown in Figure 4A. The analysis revealed a 1:1 kinetic with an equilibration constant *K*_D_ of 10.99 μM at 25°C with high dissociation and association rates. The equilibration curve analysis resulted in a similar *K*_D_ of 11.99 ± 0.00 μM (Supplementary Figure S7A), suggesting a low affinity of CrcA for Crc. As a positive control, we used different concentrations of the Hfq/*amiE*_6ARN_ protein/RNA complex (Figure 4B) that interacts with Crc (Sonnleitner et al., 2018; Pei et al., 2019). The determined *K*_D2_ value (112.310 nM; Figure 4B) is comparable with the dissociation constant revealed in solution by microscale thermophoresis (134.6 nM) (Sonnleitner et al., 2018). The best fitted binding kinetic for the Hfq/*amiE*_6ARN_/Crc complex deviated from the ideal 1:1 kinetic, which is often observed in surface binding experiments and mainly due to mass transport limitations or heterogeneity of the surface/binding sites (Schuck and Zhao, 2010; Zhao and Schuck, 2017). As Crc is immobilized on the surface, it is possible that not all interaction sites were available for multivalent binding of Crc to Hfq/*amiE*_6ARN_ (Pei et al., 2019), which might explain the observed heterogeneous binding affinities. Hfq in the absence of RNA (Supplementary Figure S7B) and ribosomal protein S1 (Supplementary Figure S7C) were further used as negative controls, and as anticipated, showed no interaction with Crc.

In addition, CrcA was immobilized on the chip surface and serial dilutions of Crc protein were added (from 20 μM to 156.25 nM; Figure 4C). The kinetic evaluation fitted best to a heterogeneous ligand model. This might be caused by the high CrcA-ligand concentration of 9.5 ng/mm^2^ on the chip surface and a potential heterogeneity in binding site availability. Nevertheless, the dissociation constant *K*_D1_ of 8.353 μM (Figure 4C) is in agreement with the calculation from the equilibration curve (7.141 ± 0.003 μM; Supplementary Figure S7D) and comparable with the results of CrcA bound to the immobilized Crc protein (Figure 4A; Supplementary Figure S7A). As expected, neither the Hfq/*amiE*_6ARN_ complex (Figure 4D) nor Hfq and S1 protein (Supplementary Figures S7E,F), respectively, were able to bind the immobilized CrcA protein. In summary, although these biophysical assays further support a direct interaction between CrcA and Crc, it appeared to be weak under the employed experimental conditions.

### Prolonged lag phase in the absence of CrcA upon relief of CCR

**Figure 5:**
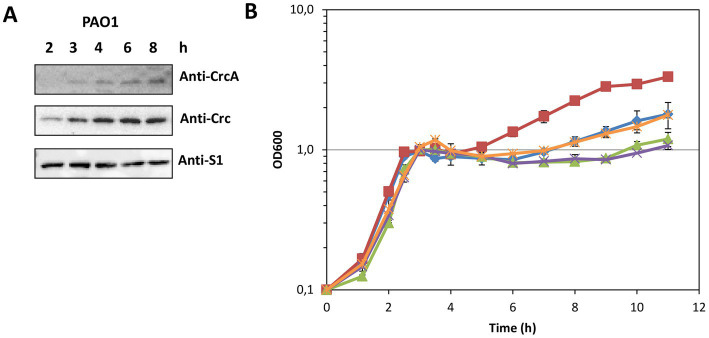
**(A)** Accumulation of CrcA upon depletion of succinate during diauxic growth of strain PAO1 in BSM supplemented with 5 mM succinate (preferred C-source) and 40 mM mannitol (less-preferred C-source). Samples were withdrawn from the PAO1 culture during growth (see **B**, blue graph marked with diamonds) at the times indicated. Equal protein concentrations of the samples were loaded onto a 12.5% SDS-polyacrylamide gel. The protein levels of CrcA, Crc and S1 (control) were determined by quantitative western-blotting using anti-CrcA, anti-Crc and anti-S1 antibodies. **(B)** Prolonged lag phase in the absence of CrcA upon diauxic growth in BSM supplemented with succinate and mannitol after depletion of succinate. The strains PAO1Δ*crc* (red squares), PAO1 (blue diamonds), PAO1ΔPA*1677* with an in frame deletion of the *crcA* gene (green triangles), PAO1ΔPA*1677*(pMMB67HE) (vector control; purple crosses) and strain PAO1ΔPA*1677*(pMMB-Strep-*1677*) (ectopic expression of *strep*-*crcA*; orange stars) were grown in BSM as specified in **(A)** and diauxic growth was monitored by measuring the optical density of the cultures at 600 nm.

The results shown in Figure 1 suggested that CrcA titrates Crc, and thereby diminishes translational repression of Hfq/Crc target genes. A relief of CCR is known to be mediated through titration of Hfq by the CrcZ RNA (Sonnleitner and Bläsi, 2014; Moreno et al., 2015), which is predominantly synthesized during the lag phase of diauxic growth, e.g., after depletion of succinate in BSM supplemented with succinate and mannitol (Rozner et al., 2022). Therefore, we asked whether CrcA might likewise accumulate under the same conditions. The *in vivo* synthesis of CrcA was assessed in strain PAO1 during diauxic growth in the presence of 5 mM succinate and 40 mM mannitol by employing quantitative Western-blotting. As shown in Figures 5A,B, the CrcA levels increased during the lag phase and after growth resumed on mannitol.

We hypothesized that the accumulation of CrcA during the lag phase of diauxic growth might lead to a titration of Crc, i.e., to a diminished *de novo* formation of repressive Hfq/Crc complexes, and thus contribute to a swifter translation of transcripts encoding functions required for utilization of non-preferred carbon sources. Therefore, we compared the diauxic growth profiles of strains PAO1Δ*crc*, PAO1, and PAO1ΔPA*1677* (*ΔcrcA*) in BSM supplemented with 5 mM succinate and 40 mM mannitol. As shown in Figure 5B, exponential growth on succinate (CCR in place) was analogous for all three strains. During CCR, genes required for the uptake and utilization of mannitol (e.g., PA*2338* and *mtlD*) are known to be repressed by Hfq/Crc (Rozner et al., 2022). After depletion of succinate, the three strains entered the lag-phase after which growth resumed on mannitol (CCR relief; Figure 5B). Owing to the function of Crc as a co-repressor in Hfq/Crc repressive complexes, i.e., its contribution to the repression of functions required for mannitol utilization (Sonnleitner et al., 2018; Rozner et al., 2022), the strain PAO1Δ*crc* displayed a shorter lag phase and a faster resumption of growth on mannitol than the parental strain PAO1 (Figure 5B). However, the strain PAO1ΔPA*1677* (Δ*crcA*) showed an extended lag phase and a delayed resumption of growth on mannitol when compared to the parental strain PAO1. Ectopic expression of *strep-crcA* in strain PAO1ΔPA*1677*(pMMB-Strep-*1677*) mirrored the diauxic growth profile of PAO1, whereas the strain PAO1ΔPA*1677*(pMMB67HE) (vector control) behaved as PAO1ΔPA*1677* (Figure 5B). Hence, the phenotype displayed by strain PAO1ΔPA*1677* (*ΔcrcA*) supported our hypothesis that CrcA binds to and titrates Crc.

## Discussion

In this study, we have identified CrcA as an interacting partner of Crc. According to bioinformatic predictions (Winsor et al., 2016) CrcA belongs to the isochorismatase-like hydrolase superfamily. A comparison of the three dimensional structure of CrcA (Figure 2) with predicted structural models of *Pae* and *E.coli* proteins generated by AlphaFold (Jumper et al., 2021), using the DALI server (Holm and Sander, 1996; Holm and Park, 2000) supported these predictions by showing the strongest matches with members of the isochorismatase-like superfamily (Supplementary Table S6). In *Pae*, PhzD (Figure 2) is a member of this family and known to be involved in phenanzine biosynthesis (Parsons et al., 2003). Other members of the family include *E. coli* RutB (Figure 2), involved in pyrimidine degradation (Kim et al., 2010), EntB, required for the synthesis of the siderophore enterobactin (Gehring et al., 1997), and PncA, involved in NAD+ synthesis (Pardee et al., 1971; Supplementary Table S6). Another group with structural homology to the isochorismate-like family are proteins of *Leishmania* (Figure 2) and *Trypanosoma*. These were annotated as mitochondrial-associated endoribonuclease Mar1 on the basis of biochemical characterization of the close homolog of *Leishmania tarentolae* (Alfonzo et al., 1998; Caruthers et al., 2005). As the formal possibility existed that CrcA is endowed with an RNase activity, we hypothesized that this activity could result in a breakdown of Hfq/Crc/RNA assemblies during the lag phase of diauxic growth. We envisioned that this could also affect the duration of the lag phase during diauxic growth as seen with strain PAO1ΔPA*1677* (Figure 5). Therefore, we made several efforts (not shown) to test whether CrcA is an RNase. However, under all conditions tested and with all RNA substrates used, we were unable to attribute such an activity to CrcA.

CrcA co-purified with and bound to Crc (Figures 3, 4). Nevertheless, the GCI assays (Figure 4) indicated that the Crc-CrcA interaction is weak. Interestingly, higher-order multi-enzyme complexes were previously described for *E. coli* EntB (Khalil and Pawelek, 2009; Pakarian and Pawelek, 2016; Ouellette et al., 2022). The interaction of EntB and EntE occurred *in vitro* with low affinity in the μM range (Khalil and Pawelek, 2009), similar as seen with CrcA and Crc (Figure 4). However, binding of the substrate 2,3-dihydroxybenzoic acid to EntE and EntB primed these proteins for efficient complexation (Khalil and Pawelek, 2009). In this regard, it is conceivable that a CrcA ligand-present or being modified during the lag phase of diauxic growth-might enhance the interaction of CrcA and Crc. Using proteomics and metabolomics, we are currently searching for such a hypothetical factor.

CrcA protein accumulates during the lag phase of diauxic growth (Figure 5A). Although the underlying molecular mechanism(s) remain to be elucidated, we have excluded that the up-regulation of the *crcA* gene is due to an upstream promoter with a σ^S^-like signature (not shown). The presence of CrcA resulted in specific de-repression of Hfq/Crc controlled genes (Table 1; Figure 1). Therefore, we also tested whether CrcA might impact on Hfq/Crc/(target)RNA assemblies, i.e., whether CrcA binding to Crc in such complexes might lead to its disassembly. This *modus operandi* could also explain the observed de-repression of Hfq/Crc target genes (Figure 1). However, *in vitro* electrophoretic mobility shift assay competition experiments with preformed Hfq/Crc/RNA complexes and purified CrcA did not support this scenario (not shown).

**Figure 6:**
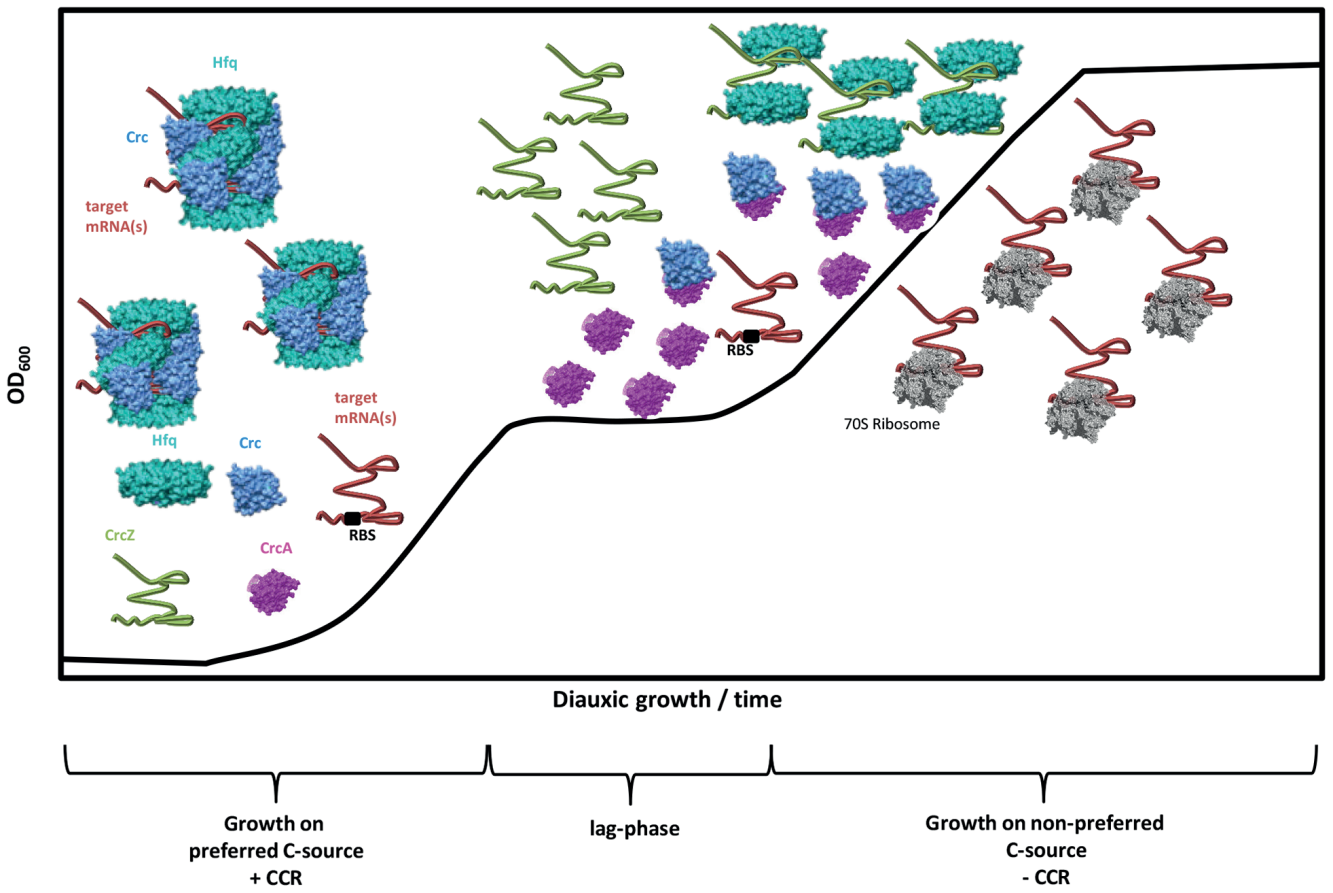
Working model for the *modus operandi* of CrcA during diauxic growth. During CCR (+CCR) and growth on a preferred C-source, e.g., succinate, the translation of target mRNA(s) encoding functions for the breakdown of less preferred C-sources are repressed by Hfq/Crc assemblies that shield the ribosome binding site (RBS). During CCR, the levels of CrcA and CrcZ RNA are low. After depletion of the preferred C-source, and upon entering the lag-phase, both, the levels of CrcA and the CrcZ RNA increase. Owing to the high affinity of Hfq for CrcZ, the RNA titrates Hfq. At the same time, CrcA titrates Crc, which prevents assembly of Crc into repressive Hfq/Crc/(target)RNA complexes. Consequently, titration of Hfq and Crc by the CrcZ RNA and CrcA, respectively, will permit translation of “catabolic genes” that were translationally silenced by Hfq/Crc during CCR, leading to metabolization of less preferred C-source(s), and continued growth after CCR is relieved (-CCR).

Taken together, our studies suggest the following working model for and function of CrcA in CCR of *Pae* (Figure 6). During CCR in the presence of a preferred C-source, e.g., succinate, and during logarithmic growth, the translation of mRNAs encoding functions for the breakdown of less preferred C-sources are translationally silenced by Hfq/Crc repressive complexes (Sonnleitner and Bläsi, 2014; Dendooven et al., 2023). As long as CCR is in place, the levels of CrcA (Figure 5B) and CrcZ RNA are low (Rozner et al., 2022). After depletion of the preferred C-source, and upon entering the lag-phase, the levels of both, CrcA and the CrcZ RNA increase. Owing to the high affinity of Hfq for CrcZ, the RNA titrates Hfq (Sonnleitner and Bläsi, 2014; Sonnleitner et al., 2018), and perhaps Hfq/Crc complexes (Moreno et al., 2015). In addition, CrcA titrates Crc, which prevents the assembly of Crc into repressive Hfq/Crc/(target)RNA complexes. Consequently, titration of Hfq and Crc by the CrcZ RNA and CrcA, respectively, will permit translation of “catabolic genes” that were subject to translational repression by Hfq/Crc during CCR, leading to metabolism of less preferred C-source(s), and continued growth. Hence, a two-pronged strategy appears to be in place to abrogate CCR; that is the established sequestration of Hfq by the RNA CrcZ and the titration of Crc by CrcA.

## Data availability statement

The datasets presented in this study can be found in online repositories. The names of the repository/repositories and accession number(s) can be found in the article/Supplementary material.

## Author contributions

ES, FB, IM, BFL, and UB conceived and designed the experiments. ES, FB, LD, PB, and AR performed the experiments. ES, BL, FB, ACS, BFL, IM, and UB analyzed the data. ES and UB wrote the paper. All authors contributed to the article and approved the submitted version.

## Funding

The work was supported by the Austrian Science Fund (FWF; www.fwf.ac.at/en) through projects P28711-B22 (UB and ES) and P26946-B20 (IM). ACS and BL were supported through the FWF funded doctoral program RNA-Biology W-1207. BFL was supported by a Wellcome Trust Investigator award (200873/Z/16/Z).

## Conflict of interest

The authors declare that the research was conducted in the absence of any commercial or financial relationships that could be construed as a potential conflict of interest.

## Publisher’s note

All claims expressed in this article are solely those of the authors and do not necessarily represent those of their affiliated organizations, or those of the publisher, the editors and the reviewers. Any product that may be evaluated in this article, or claim that may be made by its manufacturer, is not guaranteed or endorsed by the publisher.

## Footnotes

^1^ http://www.csf.ac.at

^2^ http://www.bioinformatics.babraham.ac.uk/projects/fastqc/
