# Supplementary material for: Catabolite repression control protein antagonist, a novel player in Pseudomonas aeruginosa carbon catabolite repression control
Source: Front Microbiol. 2023 May 12;14:1195558. doi: 10.3389/fmicb.2023.1195558 (PMC10213629; doi:10.3389/fmicb.2023.1195558)
Supplement: Supplementary file 1 [file Data_Sheet_1.pdf]

Supplementary Material

Catabolite repression control protein antagonist, a novel player in *Pseudomonas aeruginosa* carbon catabolite repression control

Elisabeth Sonnleitner[1*,#], Flavia Bassani[1#], Anastasia Cianciulli Sesso[1,2], Paul Brear[3], Branislav Lilic[1,2], Lovro Davidovski[1], Armin Resch[1], Ben F. Luisi[3], Isabella Moll[1] and Udo Bläsi[1*]

[1]Department of Microbiology, Immunobiology and Genetics, Max Perutz Labs, Center of Molecular Biology, Vienna Biocenter, University of Vienna, Dr. Bohrgasse 9/4, 1030 Vienna, Austria. [2]Vienna BioCenter PhD Program, a Doctoral School of the University of Vienna and the Medical University of Vienna, Max Perutz Labs, Center of Molecular Biology, Vienna Biocenter, University of Vienna, Vienna, Austria. [3]Department of Biochemistry, University of Cambridge, Cambridge, United Kingdom

*To whom correspondence should be addressed.

Udo.Blaesi@univie.ac.at

Elisabeth.Sonnleitner@univie.ac.at

[#] Equal contribution

1 Supplementary Text

Supplementary Materials and Methods

Construction of Strain PAO1ΔPA*1677* (PAO1Δ*crcA*)

The deletion of the coding sequence of PA*1677* (*crcA*) (coordinates 1827225 - 1827821 of the PAO1 genome; Winsor et al., 2016) was achieved by homologous recombination (Zhang et al., 2017). Briefly, plasmid pEXG2-ΔPA*1677* (see below) was mobilized into strain PAO1 with the aid of *E. coli* strain S17-1, and then chromosomally integrated through selection for gentamicin. Excision of the vector by a second crossover event was achieved by selection of sucrose insensitive cells as the pEXG2 vector encodes the *Bacillus subtilis sacB* gene, whose gene product – levan sucrase – renders Gram-negative cells sensitive to sucrose (Rietsch et al., 2005; Hmelo et al., 2015).

Construction of Plasmids

Plasmid pEXG2-ΔPA*1677*. To construct an in frame deletion of PA*1677* (*crcA*) the following procedure was used. Two PCR products flanking the PA*1677* gene were obtained from PAO1 chromosomal DNA with primer pairs Q181/R181 (Q181: 5'- AAA AGG ATC CTT GGA CAC GAT CAG GTC GAA GCG CTG; R181: 5'- GTC ACT ACT CCT GAG AAG GCA CAT GCC-3') and S181/T181 (S181: 5'- TGT GCC TTC TCA GGA GTA GTG ACT GAC CGC CCA TCC AGC CGC-3'; T181: 5'- AAA AGA ATT CAC GTC GAT GAC GAA CTG GTC GGA C-3'), respectively. The combined 769-bp upstream and 740-bp downstream fragments were used as a template for a second overlapping PCR with primers Q181 and T181, which was possible because the primers R181 and S181 carry a complementary sequence. The resulting fragment with a 594-bp

deletion, which spans the entire PA*1677* coding region except for the stop codon, was cleaved with *Bam*HI and *Eco*RI, and ligated into the corresponding sites of the vector pEXG2.

Plasmid pMMB-Strep-*crc*. A DNA fragment containing the full-length coding region of the Crc protein of strain PAO1 was amplified by PCR using pETM14lic-His_6Crc as template and oligonucleotides N163 (5′-TTT TTG CTA GCA TGC GGA TCA TCA GTG TGA ACG TGA-3′) and L5 (5′-ATG CCT GCA GTC AGA TGC TCA ACT GCC AG-3′). The PCR product was cleaved with *Nhe*I and *Pst*I, and ligated into the corresponding sites of plasmid pBGG237 to generate a fusion protein between a Strep-tag II and the N-terminus of Crc. This plasmid was used as a template for PCR amplification with the oligonucleotides O163 (5′-TTT TAA GCT TAG ATC TTA ATA ATT TTG TTT AAC TTT AAG AAG GAG ATA TAC ATA TGT GG-3′) and L5. The PCR product was digested with *Hind*III and *Pst*I, and ligated into the corresponding sites of plasmid pMMB67HE.

Plasmid pMMB-Strep-*1677*. The coding region of PA*1677* was PCR-amplified using the oligonucleotides W181 (5′- AAA AAG CTA GCA TGC CTC ATC CGC TCA CCC TTT TGC AG-3′) and X181 (5′-AAA AGG ATC CTC AGA GCA GTT CCC GGG CCT GGG-3′) and chromosomal DNA of strain PAO1 as template. The PCR fragment was digested with *Nhe*I and *Bam*HI, and ligated into the corresponding sites of pMMB-Strep-*crc*, exchanging the *crc* encoding gene with PA*1677* fused to the Strep-tag.

Plasmid pMMB-Strep-*3919*. The coding region of PA*3919* was PCR-amplified using the oligonucleotides B186 (5′- AAA AGC TAG CAT GGA TGA CCA CGG ACG TTC CC -3′) and C186 (5′- AAA AGG ATC CTC ACA TGT GCG CCT CGG CGT AC -3′) and chromosomal DNA of strain PAO1 as template. The PCR fragment was digested with *Nhe*I and *Bam*HI, and ligated into the corresponding sites of pMMB-Strep-*crc*, exchanging the *crc* encoding gene with PA*3919* fused to the Strep-tag.

Plasmid pMMB-Strep-*1902*. The coding region of PA*1902* (*phzD2*) was PCR-amplified using the oligonucleotides Y185 (5′- AAA AGC TAG CAT GAG CGG CAT TCC CGA AAT CAC -3′) and Z185 (5′- AAA AGG ATC CTC ATT CCA GCA CCT CGT CGG -3′) and chromosomal DNA of strain PAO1 as template. The PCR fragment was digested with *Nhe*I and *Bam*HI, and ligated into the corresponding sites of pMMB-Strep-*crc*, exchanging the *crc* encoding gene with PA*1902* fused to the Strep-tag.

Plasmid pMMB-His-3C-*crc*. A DNA fragment containing the full-length coding region of Crc protein of PAO1 was amplified by PCR using pETM14lic-His_6Crc as template and the oligonucleotides W185 (5′-TTTTAAGCTTAATAATTTTGTTTAACTTTAAGAAGGAGATATA -3′) and X4_crcrev (5′-ATGCGGATCCTCAGATGCTCAACTGCCAG -3′). The PCR product was cleaved with *Hind*III and *Bam*HI, and ligated into the corresponding sites of the expression plasmid pMMB67HE. In the resulting plasmid pMMB67HE-His-3C-*crc*, the *crc* gene is under transcriptional control of a T7 promoter and inserted in a manner that the corresponding protein is fused to an N-terminal cleavable His_6-tag.

Plasmid pMMB-His-3C-*1677*. First, the His_6-tag, containing the GST-HRV14-3C “PreScission” cleavage site (3C) and an in frame *Nde*I restriction site, was amplified with the oligonucleotides Y99 (5’- TAA TAC GAC TCA CTA TAG -3’) and D186 (5’- AAA AGG ATC **CC**T GCA GTT ACT ACA TAT GGG GCC CCT GGA ACA GAA CTT C -3’), using plasmid pETM14lic-His_6Crc as template. The PCR fragment was cleaved with *Xba*I and *Bam*HI, and ligated into the corresponding sites of plasmid pET28, generating pET28-His6-3C-NdeI. The PA*1677* coding region was amplified by PCR with the oligonucleotides E186 (5’- AAA AAC ATA TGC CTC ATC CGC TCA CCC TTT TGC AG -3’) and X181 (5’- AAA AGG ATC CTC AGA GCA GTT CCC GGG CCT GGG 3’) and chromosomal DNA of strain PAO1, then digested with *Nde*I and

*Bam*HI, and ligated into the corresponding sites of plasmid pET28-His6-3C-NdeI. The resulting plasmid pET28NdeI-PA*1677* was used as template for PCR with oligonucleotides W185 (5'- TTT TAA GCT TAA TAA TTT TGT TTA ACT TTA AGA AGG AGA TAT A -3') and X181 to amplify the entire region of His6-3C-PA*1677* abutted to the *rbs* of T7 gene 10. The PCR fragment was cleaved with *Hind*III and *Bam*HI and ligated into the corresponding sites of plasmid pMMB67HE, resulting in pMMB-His-3C-*1677*.

Plasmid pME6015-PA*2338*. To construct a translational gene fusion between PA*2338* and *lacZ* a 177-bp fragment (nt –156 to nt +21 with regard to the A (+1) of the start codon) including the PA*2338* promoter was amplified by PCR using the oligonucleotides E88 (5'-TTT TT**G** AAT TCC CTC CAG CGC CCC ACC-3') and F88 (5'-TTT TTC TGC AGG GCC TTG ATC GAG TCG TTC AT-3') and chromosomal DNA of PAO1 as template. The PCR fragment was cleaved with *Eco*RI and *Pst*I, and then ligated into the corresponding site of plasmid pME5015, abutting the 7th codon of PA*2338* to the 8th codon of *lacZ*.

Mass spectrometry Data Analysis

All proteins present in the mock control and those co-purifying with Strep-Crc were analyzed by mass spectrometry: A 100 µl aliquot of each eluate was denatured in 4 M urea, 50 mM ammonium bicarbonate (ABC), before reducing the disulfide bonds with 10 mM dithiothreitol for 30 min at room temperature. Free thiols were alkylated in the presence of 20 mM iodoacetamide in the dark, and the solution was then diluted with 50 mM ABC to 1 M urea. The proteins were treated overnight at 37 °C with trypsin (Promega, Trypsin Gold) at a ratio of 1:50 of trypsin to protein. The reaction was stopped with trifluoroacetic acid, and the peptides were desalted on C18 Stage tips (Rappsilber et al., 2007). The tryptic digests were separated on an Ultimate 3000 RSLC nano-flow chromatography

system (Thermo Fisher Scientific) using a pre-column for sample loading (PepMapAcclaim C18, 2 cm × 0.1 mm, 5 µm, Dionex-Thermo-Fisher) and a C18 analytical column (PepMapAcclaim C18, 50 cm × 0.75 mm, 2 µm, Dionex-Thermo-Fisher), applying a linear gradient from 2% up to 35% acetonitrile in 0.1% formic acid at a flow rate of 230 µl min^{-1} over 120 min. The peptides were analyzed on a Q Exactive HF Orbitrap mass spectrometer (Thermo Fisher Scientific), equipped with a Proxeon nanospray source (Thermo Fisher Scientific), operated in a data-dependent mode. Survey scans were obtained at a mass range of 380–1650 m/z with lock mass on, with a resolution of 120.000 at 200 m/z. The 10 most intense ions were selected with an isolation width of 2 Da, fragmented in the HCD cell at 27% collision energy, and the spectra were recorded at a resolution of 30000. Peptides were excluded from fragmentation with a charge of +1 and higher than 6. The peptide match and exclude isotope features were enabled and selected precursors were dynamically excluded from repeated sampling for 30 s. The raw data were processed using the MaxQuant software package (version 1.5.5.1, http://www.maxquant.org) (Cox and Mann, 2008) and searched against the PAO1 Uniprot database (http://www.uniprot.org). The search was performed with full trypsin specificity and a maximum of two missed cleavages. Cysteine carbamidomethylation (CAM) of residues was set as fixed, oxidation of methionine, N-terminal protein acetylation as variable modifications and all other parameters were set to default. The results were filtered at a protein and peptide false discovery rate of 1% and LFQ (label free quantification) was used to quantify proteins relatively between the two samples.

SEC-MALS Analysis

For the SEC-MALS (*s*ize-*e*xclusion *c*hromatography coupled to *m*ulti-*a*ngle *l*ight *s*cattering) analysis, 50 µl of purified Strep-CrcA (1.5 mg/ml) were applied to a Superdex 75 column equilibrated with 50 mM Tris-Cl pH 7.5, 150 mM NaCl. The eluant was analysed with a DAWN HELEOS II MALS detector (Wyatt Technology), followed by an Optilab T-rEX differential refractometer (Wyatt Technology) at 25 °C. Data collection and analysis was performed using ASTRA 6 software (Wyatt Technology). Bovine serum albumin was used as a standard to confirm system performance. The molecular masses were calculated from Zimm plots, using a dn/dc value of 0.1850 mL/g.

2 Supplementary Figures and Tables

2.1 Supplementary Tables

Supplementary Table S1. Strains and Plasmids used in this study.

Strains	Genotype/Relevant features	Reference
P. aeruginosa		
PAO1	wild-type	(Holloway et al., 1979)
PAO1Δ*crc*	PAO6673	(Sonnleitner et al., 2009)
PAO1Δ*hfq*		(Sonnleitner et al., 2017)
PAO1ΔPA*1677* (Δ*crcA*)		This study
E. coli		
JW4130	[F'*proAB lacI^q lacZ*ΔM15::Tn*10*; Tc^r]	(Malecka et al., 2021)
S17-1	*pro, res$^-$ hsdR17 (rK$^-$ mK$^+$) recA$^-$ RP4-2-Tc::Mu-Km::Tn7*, Tp^R	(Simon et al., 1986)
**Plasmids**		
pETM14lic-His$_6$Crc	Encodes His-tagged Crc	(Milojevic et al., 2013)
pBGG237	Strep-tag encoding sequence under transcriptional control of P_{tac}	(Lüttmann et al., 2012)
pMMB67HE	IncQ expression vector carrying an inducible P_{tac} promoter; Ap/Cb^R	(Fürste et al., 1986)
pMMB-Strep-*crc*	pMMB67HE derivative encoding the 5´ Strep-tagged Crc protein. The corresponding gene is under transcriptional control of the P_{tac} promoter; Ap/Cb^R	This study
pMMB-Strep-*1677*	pMMB67HE derivative encoding the 5´ Strep-tagged PA1677 (CrcA) protein. The corresponding gene is under transcriptional control of the P_{tac} promoter; Ap/Cb^R	This study
pMMB-Strep-*3919*	pMMB67HE derivative encoding the 5´ Strep-tagged PA3919 protein. The corresponding gene is under transcriptional control of the P_{tac} promoter; Ap/Cb^R	This study
pMMB-Strep-*1902*	pMMB67HE derivative encoding the 5´ Strep-tagged PA1902 protein. The corresponding gene is under transcriptional control of the P_{tac} promoter; Ap/Cb^R	This study
pMMB-His-3C-*crc*	pMMB67HE derivative encoding the N-terminal cleavable (3C) His$_6$-tagged Crc protein. The corresponding gene is under control of the P_{tac} promoter; Ap/Cb^R	This study
pMMB-His-3C-*1677*	pMMB67HE derivative encoding the N-terminal cleavable (3C) His$_6$-tagged PA1677 (CrcA) protein. The corresponding	This study

	gene is under control of the P_{tac} promoter; Ap/Cb^R	
pHfqPae	pUC19 derivative carrying the PAO1 *hfq* gene under transcriptional control of P_{lac} and translational control of the T7 gene 10 rbs.	(Sonnleitner and Bläsi, 2014)
pEXG2	ColE1 suicide vector; mob *sacB* GmR	(Rietsch et al., 2005)
pEXG2-ΔPA*1677*	pEXG2 with flanking regions to create an unmarked PA*1677* deletion	This study
pME9655	pME6013 carrying a translational *amiE::lacZ* fusion gene; Tc^R	(Sonnleitner et al., 2009)
pME6015	Cloning vector for translational *lacZ* fusions; Tc^R	(Schnider-Keel et al., 2000)
pME6015-PA*2338*	pME6015 carrying a translational *PA2338::lacZ* fusion gene	This study

Supplementary Table S2. Summary of Crystallographic Data Collections.

Space group	cell	Crystallisation condition	Strep.CrcA in asymmetric unit	Resolution	Rwork/Rfree*
**$C222_1$**	**85.28 245.26 126.26**<br>**90 90 90**	**H8**	**3 dimers**	**1.9**	**0.35**
**I432**	**155.71 155.71 155.71**	**F11**	**1 Strep-CrcA**	**5.9**	
**$P6_2$**	**93.20 93.20 135.41**<br>**90 90 120**	**C11**	**1 dimer**	**1.9**	**0.47**
**$P4_22_12$**	**95.03 95.03 86.04 90 90 90**	**H4**		**1.8**	
**P4 (expanded to P1)**	**54.71, 87.7001, 87.7001, 84.4168, 71.8253, 71.8253**	**F6 classics**	**4 dimers**	**2.3**	**0.32**
**R3 (hexagonal setting)**	**220.00 220.00 118.92 90 90 120**	**C2 BCS**		**2.5**	

*Rwork/Rfree is from PHASER solutions using the refined dimer and monomer from the model refined against the $C222_1$ data set (see **Supplementary Table S3**).

Supplementary Table S3. Refinement Parameters for the C222$_1$ Crystal of Strep-CrcA.

PDB code	8CIB
Beamline	Diamond beamline i24
Wavelength	0.9999
Resolution range	122.63 - 1.78 (1.88 - 1.78)
Space group	C 2 2 21
Unit cell	85.284 245.26 126.262 90 90 90
Total reflections	845570 (58756)
Unique reflections	120918 (13457)
Multiplicity	7.0 (4.4)
Completeness (%)	96.2 (73.9)
Mean I/sigma(I)	6.7 (1.3)
Wilson B-factor	19.43
R-merge	0.24 (1.21)
R-meas	0.29 (1.49)
R-pim	0.15 (0.84)
CC1/2	0.99 (0.42)
Refinement	
Resolution range	122.63-1.78 (1.83-1.78)
Reflections used in refinement	120445 (8644)
Reflections used for R-free	6093 (438)
R-work	0.17 (0.31)
R-free	0.21 (0.33)
Number of non-hydrogen atoms	9892
macromolecules	8883
ligands	52
solvent	957
Protein residues	1179
RMS(bonds)	0.014
RMS(angles)	1.77
Ramachandran favored (%)	97.17
Ramachandran allowed (%)	2.83
Ramachandran outliers (%)	0
Rotamer outliers (%)	1.42
Clashscore	2.61
Average B-factor	24.48
macromolecules	23.4
ligands	38.99
solvent	33.7

Supplementary Table S4. Putative Crc Interaction Partners identified after Strep-tactin® Co-purification and LC-MS/MS analysis.

Protein	Mol. weight [kDa]	Sequence coverage [%]	MS/MS count CRC (BSM complex)	MS/MS count Mock (BSM complex)	Function
Strep-Crc	31,174	86,3	1403	1	catabolite repression control protein
PA1677	21,034	98	35	0	conserved hypothetical protein (isochorismatase-like superfamily)
Hfq	9,1034	98,8	23	1	Hfq-protein
PA3919	51,699	53,3	10	0	conserved hypothetical protein (PhoH-like protein)

Supplementary Table S5. RNA-seq analysis of PAO1ΔPA*1677*(pMMB-Strep-*1677*) versus PAO1ΔPA*1677*(pMMB67HE) grown in BSM complex medium to an OD_{600} of 1.5. The values of the fold-change and adjusted P-values are highlighted in pink. Also highlighted are the fold-change and adjusted P-values of transcripts overlapping with the RNA-seq analysis performed by Sonnleitner et al. (2018). In the latter study the transcriptome profiles of strain PAO1*hfq*- and PAO1 (*hfq*- *vs wt*) and PAO1Δ*crc* and PAO1 (Δ*crc vs wt*) were compared. The respective fold-change and adjusted P-values of transcripts are highlighted in blue and green, respectively. Transcripts that showed an increased abundance in the absence of either *hfq* and/or *crc* are indicated in bold.

locus_tag e	gene_name	baseMean	FoldChange ov vs del	pvalue	padj	baseMean ov	baseMean del	*hfq- vs wt*	padj2	Δ*crc vs wt*	padj3	Description
PA0090	*clpV1*	1620	-2,59	8,35E-04	3,50E-02	902	2338					AAA+ ATPase ClpV1
PA0091	*vgrG1*	728	-2,30	7,83E-04	3,37E-02	442	1015	-2,0	3,1E-04			VgrG1
PA0471	*fiuR*	271	2,57	3,08E-06	1,94E-04	390	152					sigma factor regulator FiuR
PA0472	*fiuI*	337	2,48	2,02E-05	1,14E-03	480	194					sigma factor FiuI
PA0672	*hemO*	731	12,79	1,17E-32	2,55E-30	1355	106					heme oxygenase
PA0707	*toxR*	119	2,27	7,92E-04	3,37E-02	165	73					transcriptional regulator ToxR
PA0865	*hpd*	1032	2,89	5,92E-09	4,93E-07	1533	531			-2,5	7,6E-03	4-hydroxyphenylpyruvate dioxygenase
PA0931	*pirA*	1074	4,74	4,16E-21	6,52E-19	1773	374					ferric enterobactin receptor PirA
PA0985	*pyoS5*	398	2,15	1,10E-04	5,65E-03	543	252	-4,2	8,2E-04	-3,8	1,9E-03	pyocin S5
PA1134		42	9,72	3,78E-08	2,86E-06	76	8					hypothetical protein
PA1245	*aprX*	840	2,34	1,83E-05	1,05E-03	1177	503	-5,0	4,7E-28	-8,3	2,1E-65	AprX
PA1246	*aprD*	252	2,47	1,66E-05	9,69E-04	358	145	-5,0	7,1E-22	-9,1	7,1E-32	alkaline protease secretion protein AprD
PA1300	*hxuI*	171	3,09	3,13E-05	1,74E-03	259	84					HxuI
PA1301	*hxuR*	168	5,50	9,36E-11	9,15E-09	285	52					anti-sigma factor HxuR
PA1302	*hxuA*	189	2,73	1,13E-06	7,56E-05	276	101	2,9	6,3E-07			Heme receptor HxuA
PA1677		233919	8112,55	0,00E+00	0,00E+00	467780	58					putative isochorismatase
PA1713	*exsA*	1232	2,13	7,91E-04	3,37E-02	1677	786	10,6	6,1E-04			transcriptional regulator ExsA
PA1899	*phzA2*	32	3,60	3,63E-04	1,63E-02	50	14	-29,3	2,9E-08	-12,3	8,6E-06	probable phenazine biosynthesis protein
PA1911	*femR*	89	2,98	9,05E-06	5,40E-04	133	45					sigma factor regulator, FemR
PA2033		351	5,47	1,23E-20	1,83E-18	594	109					hypothetical protein
PA2034		77	4,82	3,94E-09	3,44E-07	128	27					hypothetical protein

**PA2247**	***bkdA1***	640	2,66	3,33E-10	3,14E-08	929	350	**77,6**	**6,5E-139**	**2,3**	**4,7E-10**	2-oxoisovalerate dehydrogenase (alpha subunit)
**PA2248**	***bkdA2***	449	2,64	3,69E-09	3,32E-07	650	247	**85,7**	**9,0E-143**	**2,5**	**2,3E-11**	2-oxoisovalerate dehydrogenase (beta subunit)
**PA2249**	***bkdB***	601	2,51	1,13E-08	9,12E-07	860	342	**61,2**	**4,6E-128**	**2,2**	**1,0E-08**	branched-chain alpha-keto acid dehydrogenase (lipoamide component)
**PA2250**	***lpdV***	624	2,78	5,54E-08	4,13E-06	917	330	**38,9**	**6,9E-108**	**2,0**	**8,4E-08**	lipoamide dehydrogenase-Val
**PA2338**		625	4,24	6,69E-18	9,03E-16	1012	238	**12,9**	**2,9E-62**	**10,6**	**5,0E-67**	probable binding protein component of ABC maltose/mannitol transporter
**PA2339**		86	4,97	3,29E-09	3,00E-07	143	29	**21,8**	**3,7E-82**	**19,3**	**1,2E-114**	probable binding-protein-dependent maltose/mannitol transport protein
**PA2340**		65	3,98	3,06E-06	1,94E-04	104	26	**22,7**	**4,7E-83**	**20,0**	**2,0E-115**	probable binding-protein-dependent maltose/mannitol transport protein
**PA2341**		172	5,09	5,76E-15	6,80E-13	287	56	**17,3**	**1,5E-73**	**16,2**	**1,3E-106**	probable ATP-binding component of ABC maltose/mannitol transporter
**PA2342**	***mtlD***	149	4,43	3,12E-11	3,10E-09	243	55	**23,8**	**1,1E-85**	**22,4**	**3,4E-124**	mannitol dehydrogenase
**PA2343**	***mtlY***	62	5,74	1,90E-06	1,24E-04	106	18	**21,3**	**7,3E-80**	**20,7**	**5,9E-117**	xylulose kinase
PA2384		179	3,17	1,01E-07	7,16E-06	272	86					hypothetical protein
PA2385	*pvdQ*	710	25,99	3,00E-45	1,21E-42	1367	52					3-oxo-C12-homoserine lactone acylase PvdQ
PA2386	*pvdA*	4870	34,75	4,80E-41	1,36E-38	9469	272					L-ornithine N5-oxygenase
**PA2389**	***pvdR***	370	3,69	8,61E-09	7,07E-07	581	158	**2,2**	**8,7E-06**	**2,8**	**2,6E-10**	siderophore efflux pump protein PvdR
PA2390	*pvdT*	381	4,01	2,10E-15	2,59E-13	609	152			2,1	8,3E-06	siderophore efflux pump protein PvdT
PA2391	*opmQ*	272	3,58	1,06E-12	1,11E-10	425	119					siderophore efflux pump protein OpmQ
PA2392	*pvdP*	1006	19,41	1,08E-43	4,08E-41	1913	98					pyoverdine maturaton protein PvdP
PA2393		1286	40,34	1,14E-58	9,25E-56	2510	62					putative dipeptidase
PA2394	*pvdN*	983	30,30	4,22E-41	1,28E-38	1903	62					PvdN
PA2395	*pvdO*	734	31,72	1,47E-59	1,39E-56	1423	45					PvdO
PA2396	*pvdF*	2061	15,76	1,19E-41	4,20E-39	3876	246					pyoverdine synthetase F
PA2397	*pvdE*	689	27,40	3,80E-48	1,79E-45	1330	48					pyoverdine biosynthesis protein PvdE
PA2398	*fpvA*	8619	2,04	2,66E-04	1,27E-02	11565	5674	-3,3	1,8E-06			ferripyoverdine receptor
PA2399	*pvdD*	3624	7,52	1,53E-36	3,77E-34	6398	850					pyoverdine synthetase D
PA2400	*pvdJ*	2901	7,74	1,87E-29	3,53E-27	5138	663	2,1	8,2E-07			PvdJ
PA2402	*pvdI*	7118	11,28	2,54E-40	6,54E-38	13077	1159					pyoverdine peptide synthetase
PA2411		753	21,99	1,89E-51	1,19E-48	1440	65					probable thioesterase

PA2412		735	26,68	2,75E-51	1,42E-48	1417	53					conserved hypothetical protein
PA2413	*pvdH*	994	40,50	5,05E-61	5,72E-58	1941	48					L-2,4-diaminobutyrate:2-ketoglutarate 4-aminotransferase, PvdH
PA2424	*pvdL*	3590	26,42	1,01E-55	7,14E-53	6918	261	2,1	5,4E-06			PvdL
PA2425	*pvdG*	257	25,78	3,23E-30	6,54E-28	494	19					PvdG
PA2426	*pvdS*	681	35,78	5,07E-35	1,20E-32	1325	37					sigma factor PvdS
PA2427		44	9,73	1,87E-08	1,44E-06	80	8					hypothetical protein
PA2451		608	11,29	1,39E-23	2,38E-21	1118	99					hypothetical protein
PA2467	*foxR*	308	2,34	1,57E-04	8,03E-03	431	185			2,0	5,2E-05	Anti-sigma factor FoxR
PA2468	*foxI*	402	2,26	1,68E-05	9,73E-04	557	246			2,1	2,5E-05	ECF sigma factor FoxI
PA2531		150	10,55	4,19E-23	6,99E-21	273	26					probable aminotransferase
PA2552		233	2,40	5,62E-05	3,03E-03	330	137	10,3	1,2E-03			probable acyl-CoA dehydrogenase
**PA2553**		365	2,11	2,99E-04	1,38E-02	495	234	**12,3**	**3,4E-04**	**2,1**	**2,5E-07**	probable acyl-CoA thiolase
**PA2554**		106	2,37	2,99E-04	1,38E-02	149	62	**13,1**	**7,1E-05**	**2,0**	**3,1E-04**	probable short-chain dehydrogenase
PA2555		330	2,11	4,01E-05	2,21E-03	448	212	4,1	2,5E-06			probable AMP-binding enzyme
PA2686	*pfeR*	523	2,19	3,97E-06	2,44E-04	718	328			2,2	1,4E-06	two-component response regulator PfeR
PA2687	*pfeS*	481	2,09	1,59E-04	8,06E-03	650	312			2,2	2,6E-07	two-component sensor PfeS
PA2688	*pfeA*	297	2,92	7,93E-08	5,76E-06	443	152					Ferric enterobactin receptor, outer membrane protein PfeA precursor
**PA3186**	***oprB***	182	4,29	3,19E-09	2,97E-07	296	69	**306,2**	**2,0E-179**	**164,1**	**3,0E-262**	Glucose/carbohydrate outer membrane porin OprB precursor
**PA3187**		244	9,26	5,50E-20	7,80E-18	439	48	**242,3**	**5,6E-177**	**225,3**	**5,0E-280**	probable ATP-binding component of ABC transporter
**PA3188**		49	14,32	2,85E-10	2,74E-08	91	6	**196,3**	**6,1E-174**	**196,2**	**1,5E-258**	probable permease of ABC sugar transporter
**PA3189**		69	12,67	4,09E-14	4,63E-12	129	10	**145,5**	**2,7E-165**	**127,1**	**3,0E-238**	probable permease of ABC sugar transporter
**PA3190**		1360	41,81	1,33E-74	2,51E-71	2656	63	**222,9**	**1,1E-169**	**178,9**	**2,6E-268**	probable binding protein component of ABC sugar transporter
**PA3362**	***amiS***	238	6,21	2,32E-20	3,37E-18	409	66	**32,4**	**7,3E-98**	**14,4**	**6,2E-93**	hypothetical protein
**PA3363**	***amiR***	526	10,16	1,02E-40	2,76E-38	958	94	**36,6**	**2,7E-104**	**17,3**	**1,2E-105**	aliphatic amidase regulator
**PA3364**	***amiC***	700	7,74	7,12E-35	1,61E-32	1240	160	**55,4**	**1,6E-123**	**21,7**	**3,2E-122**	aliphatic amidase expression-regulating protein
**PA3365**	***amiB***	1063	10,84	2,69E-51	1,42E-48	1947	180	**72,9**	**3,2E-136**	**26,3**	**1,1E-135**	probable chaperone
**PA3366**	***amiE***	4906	9,76	1,04E-45	4,53E-43	8901	912	**27,3**	**1,0E-93**	**8,3**	**8,8E-68**	aliphatic amidase

PA3407	*hasAp*	185	9,06	2,59E-22	4,20E-20	334	37					heme acquisition protein HasAp
PA3408	*hasR*	114	6,81	3,01E-15	3,63E-13	198	29					Heme uptake outer membrane receptor HasR precursor
PA3409	*hasS*	109	3,94	3,81E-09	3,37E-07	173	44					anti-sigma factor
PA3410	*hasI*	473	4,75	1,34E-13	1,46E-11	780	165					ECF sigma factor HasI
PA3899	*fecI*	188	3,68	8,58E-08	6,16E-06	296	81					ECF sigma factor FecI
PA3900	*fecR*	108	3,95	6,31E-07	4,26E-05	172	44					anti-sigma factor
PA3901	*fecA*	98	2,74	9,22E-06	5,44E-04	144	53					Fe(III) dicitrate transport protein FecA
**PA4022**	***hdhA***	118	3,00	3,88E-06	2,41E-04	177	59	**14,8**	**4,3E-31**	**6,1**	**5,6E-48**	hydrazone dehydrogenase, HdhA
PA4156	*fvbA*	208	3,13	1,03E-07	7,19E-06	315	101					siderophore transport protein FvbA
PA4158	*fepC*	63	2,92	2,20E-04	1,08E-02	93	32					ferric enterobactin transport protein FepC
PA4159	*fepB*	53	2,81	3,09E-04	1,41E-02	78	28					ferrienterobactin-binding periplasmic protein precursor FepB
PA4168	*fpvB*	254	15,89	6,32E-32	1,33E-29	478	30					second ferric pyoverdine receptor FpvB
PA4218	*ampP*	178	4,37	4,01E-09	3,44E-07	290	66					AmpB
PA4219	*ampO*	159	3,83	1,63E-07	1,11E-05	252	65					AmpO
PA4221	*fptA*	1623	5,36	1,40E-14	1,62E-12	2735	510					Fe(III)-pyochelin outer membrane receptor precursor
PA4225	*pchF*	716	2,72	2,50E-04	1,21E-02	1048	384					pyochelin synthetase
PA4226	*pchE*	802	3,20	3,02E-06	1,94E-04	1222	381					dihydroaeruginoic acid synthetase
PA4227	*pchR*	790	4,15	4,23E-14	4,70E-12	1273	307					transcriptional regulator PchR
PA4228	*pchD*	299	6,30	6,04E-19	8,35E-17	515	82					pyochelin biosynthesis protein PchD
PA4229	*pchC*	72	6,05	1,20E-08	9,47E-07	125	20					pyochelin biosynthetic protein PchC
PA4230	*pchB*	79	6,43	1,78E-08	1,38E-06	137	21					salicylate biosynthesis protein PchB
PA4231	*pchA*	303	5,39	2,55E-12	2,63E-10	512	95					salicylate biosynthesis isochorismate synthase
PA4467		215	9,86	2,10E-27	3,83E-25	390	40					hypothetical protein
PA4468	*sodM*	550	12,47	4,28E-41	1,28E-38	1018	81					superoxide dismutase
PA4469		331	14,42	1,41E-41	4,70E-39	619	43					hypothetical protein
PA4470	*fumC1*	1897	17,64	2,99E-67	4,24E-64	3590	203					fumarate hydratase
PA4471		151	17,95	4,26E-21	6,52E-19	286	16					hypothetical protein
PA4514		2781	2,26	1,62E-06	1,07E-04	3856	1706					probable outer membrane receptor for iron transport
PA4570		275	12,62	1,86E-24	3,30E-22	510	40					hypothetical protein

PA4707	*phuU*	400	2,70	1,20E-08	9,47E-07	583	216					Heme-transport protein PhuU
PA4708	*phuT*	802	4,95	7,25E-17	9,34E-15	1335	270					Heme-transport protein, PhuT
PA4709	*phuS*	926	9,24	1,47E-29	2,87E-27	1670	181					Heme-transport protein, PhuS
PA4710	*phuR*	3414	33,01	1,06E-86	3,01E-83	6628	201					Heme/Hemoglobin uptake outer membrane receptor PhuR precursor
PA4895		79	8,04	1,86E-13	1,99E-11	140	17					anti-sigma factor
PA4896		255	7,94	1,79E-16	2,26E-14	453	57					probable ECF sigma factor
PA4897		177	3,29	1,13E-07	7,82E-06	271	82					TonB-dependent receptor
PA5100	*hutU*	182	-2,24	1,96E-04	9,65E-03	112	252	-3,8	2,6E-03	-6,0	3,7E-04	urocanase
**PA5168**	***dctQ***	129	2,41	2,74E-04	1,29E-02	182	76	**4,8**	**1,5E-21**	**2,1**	**2,9E-06**	C(4)-dicarboxylate transport system protein DctQ
**PA5169**	***dctM***	209	3,27	4,96E-06	3,02E-04	320	98	**4,8**	**1,0E-22**	**2,2**	**3,3E-06**	C(4)-dicarboxylate transport system protein DctM
PA5445		808	-3,22	6,44E-08	4,74E-06	383	1232			-3,9	2,1E-15	probable coenzyme A transferase
PA5460		101	2,42	1,03E-04	5,34E-03	142	59					hypothetical protein

Supplementary Table S6. Three dimensional structure comparison of CrcA with predicted structural models of *E. coli* and *Pae* proteins using the Dali server.

**Protein**	**AlphaFold-ID**	**Z-score**	**% sequence identity**	**Description**
E. coli				
RutB	AF-P75897-F1	22.0	24	Ureidoacrylate amidohydrolase
EntB	AF-P0ADI4-F1	21.5	20	Enterobactin synthase component B
YecD	AF-P0ADI7-F1	21.3	25	Isochorismatase family protein
PncA	AF-P21369-F1	21.1	23	Nicotinamidase
YcaC	AF-P21367-F1	17.6	15	Isochorismatase family protein
P. aeruginosa				
PA3783	AF-Q9HXL2-F1	25.3	31	Isochorismatase family protein
PA3953	AF-Q9HX63-F1	24.4	33	Isochorismatase family protein
PhzD	AF-P0DPC1-F1	22.4	20	Phenanzine biosynthesis protein
PA5507	AF-Q9HT66-F1	21.6	27	Isochorismatase family protein
PA3066	AF-Q9HZE2-F1	21.5	24	Isochorismatase family protein

2.2 Supplementary Figures

Supplementary Figure S1

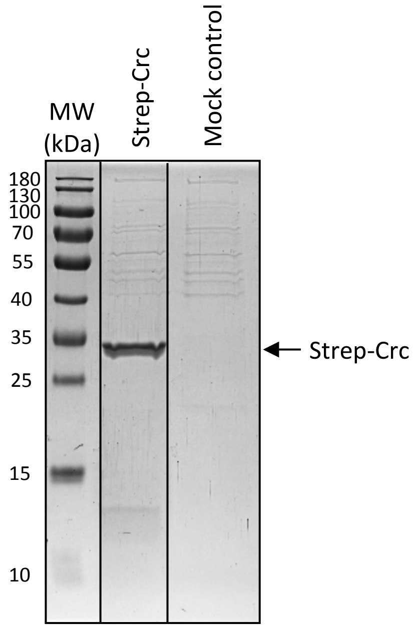

Supplementary Figure S1. Coomassie stained gel of proteins co-purifying with Strep-Crc after elution from the Strep-tactin® resin. PAO1Δ*crc*(pMMB-Strep-*crc*) (Strep-Crc) and PAO1Δ*crc*(pMMB67HE) (mock control) were grown in BSM complex medium (Sonnleitner et al., 2018) to an OD_{600} of 1.5. Then, samples were withdrawn and Strep-Crc was purified by affinity chromatography using the Strep-tactin® resin. The eluates from the mock control were obtained under the same conditions in the absence of Strep-Crc. Aliquots of the eluates used for LC-MS/MS analysis were separated using a 12.5% SDS-polyacrylamide gel. The separation of molecular weight marker proteins is shown at the left. The arrow marks the position of the Strep-Crc protein.

Supplementary Figure S2

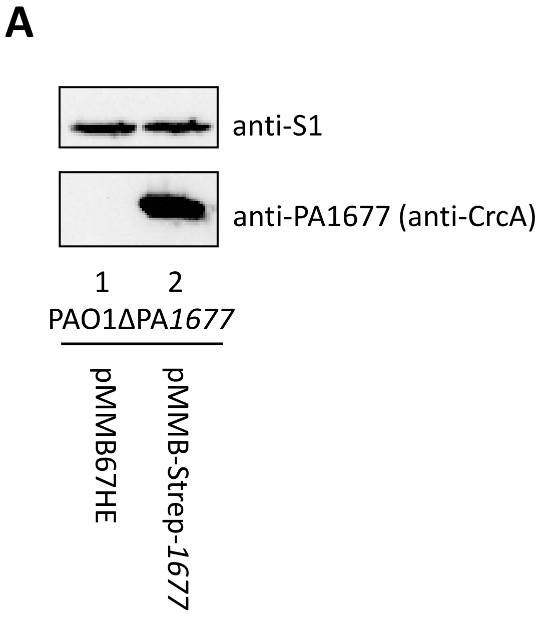

Supplementary Figure S2. Synthesis of Strep-1677 (Strep-CrcA) in strain PAO1ΔPA*1677*(pMMB-Strep-*1677*) in the presence of IPTG (1 mM final concentration). The strains PAO1ΔPA*1677*(pMMB67HE) and PAO1ΔPA*1677*(pMMB-Strep-*1677*) were grown in BSM complex medium (Sonnleitner et al., 2018) to an OD_{600} of 1.5. The Strep-PA1677 (Strep-CrcA) protein levels were determined by Western-blot analysis using anti-PA1677 (anti-CrcA) antibodies. Immunodetection of ribosomal protein S1 served as a loading control.

Supplementary Figure S3

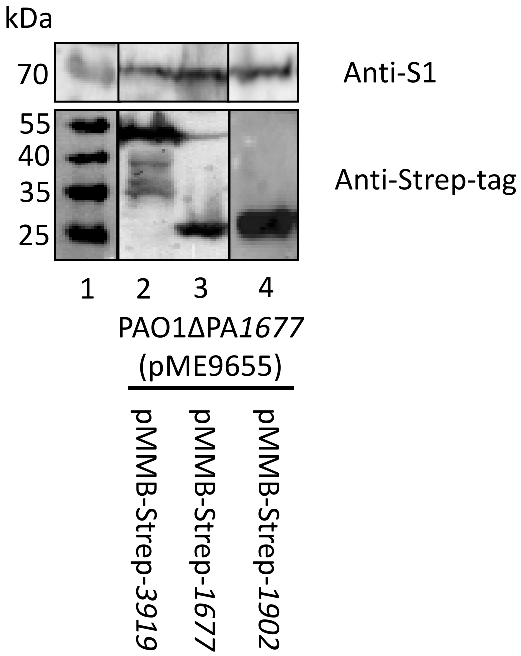

Supplementary Figure S3. Synthesis of Strep-tagged proteins. The strain PAO1ΔPA*1677*(pMMB-Strep-*3919*), PAO1ΔPA*1677*(pMMB-Strep-*1677*) and PAO1ΔPA*1677*(pMMB-Strep-*1902*) harbouring plasmid pME9655 were grown in BSM succinate to an OD_{600} of 2.0 in the presence of IPTG (1 mM final concentration). The levels of the Strep-tagged proteins were determined by Western-blot analyses using anti-Strep antibodies. Immunodetection of ribosomal protein S1 served as a loading control.

Supplementary Figure S4

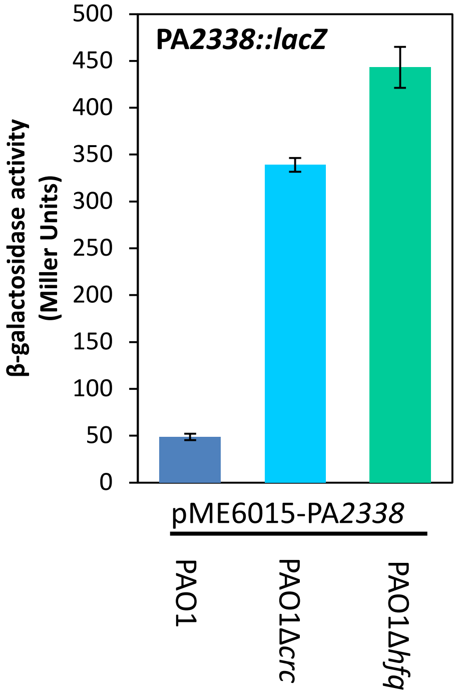

Supplementary Figure S4. Repression of the translational PA*2338::lacZ* reporter gene by Hfq and Crc. The strains were grown in BSM medium supplemented with 40 mM succinate and 5 mM mannitol to establish CCR, and to induce PA*2338::lacZ* expression. At an OD_{600} of 2.0, the cells were harvested and the β-galactosidase activities were determined. The bars depict β-galactosidase values conferred by the PA2338-LacZ fusion protein encoded by plasmid pME6015-PA*2338* in strains PAO1, PAO1Δ*crc* and PAO1Δ*hfq*. The error bars represent standard deviations from three independent experiments.

Supplementary Figure S5

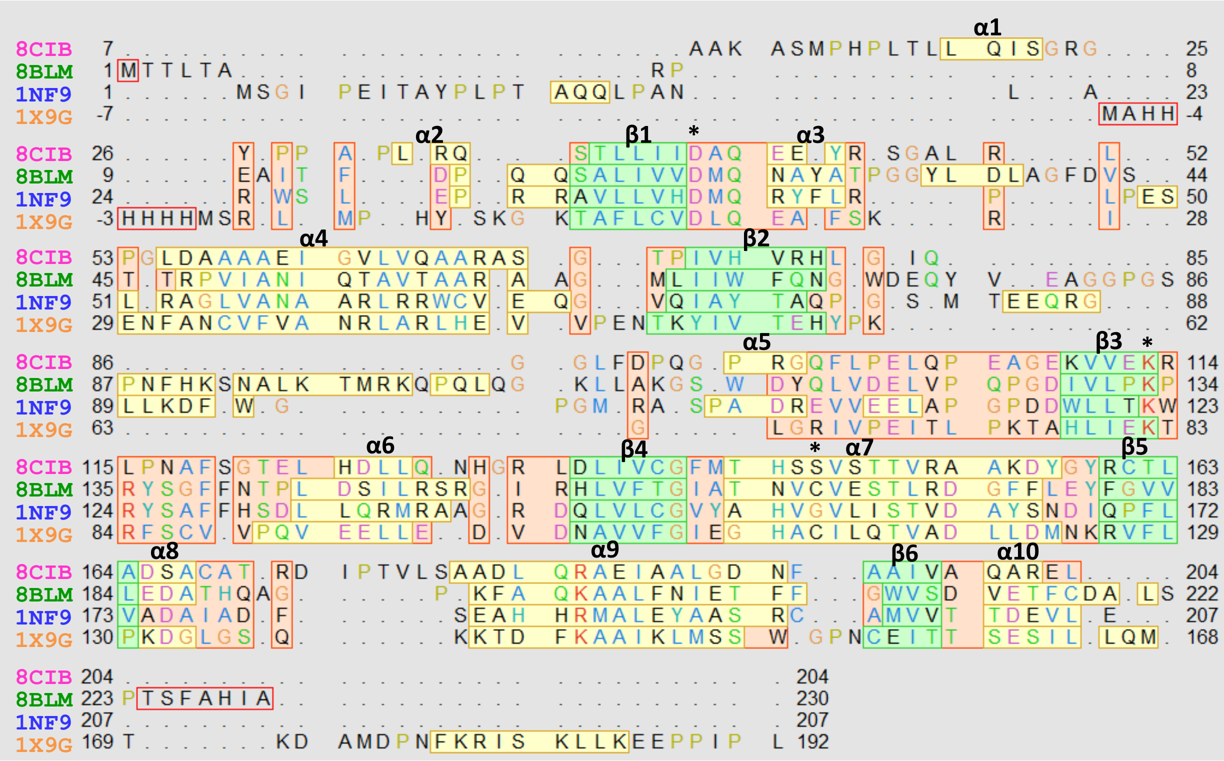

Supplementary Figure S5. Structural alignment performed with Chimera (Pettersen et al., 2004) of CrcA (8CIB), RutB (8BLM), PhzD (1NF9) and Ldon001686AAA (1X9G). Structural conservation is highlighted in orange, α-helices are highlighted in yellow and β-sheets in green. The consecutive nomenclature of α-helices and β-sheets of CrcA are shown above the alignment in accordance to **Figure 2A**. The residues of the catalytical triade are marked with an asterisk.

Supplementary Figure S6

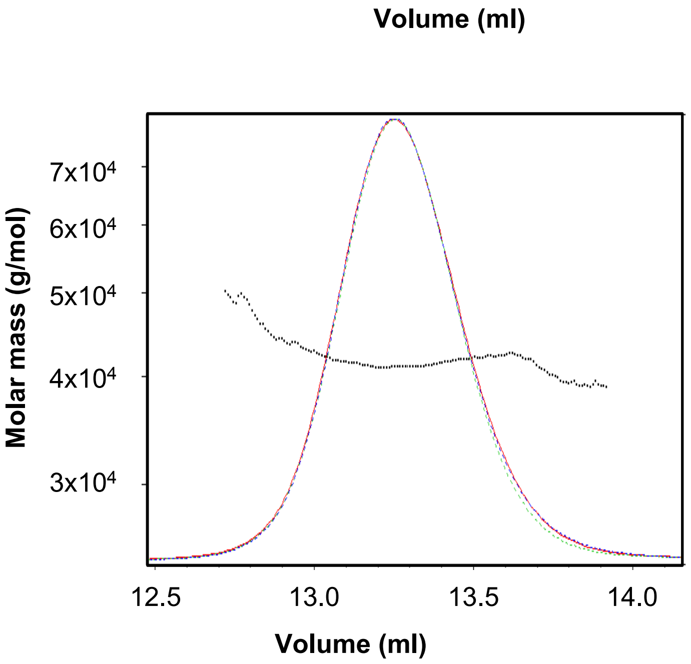

Molar mass moments (g/mol)
Mn: 4.156×10^4 (±0.599%)
Mw: 4.158×10^4 (±0.602%)
Polydispersity
Mw/Mn: 1.000 (±0.8495%)

Observed MW: 41.6kDa

Supplementary Figure S6. SEC-MALS analysis of the Strep-CrcA Dimer. The experiment was performed in 50 mM Tris-HCl pH 7.5 and 150 mM NaCl at room temperature. Left: SEC-MALS elution profile showing estimated molecular mass variation over the elution profile (red line: LS light scattering intensity, green line: UV absorbance, blue line: refractive index change, black dotted line: molar mass). Right: summary of molecular mass estimates (Mn: number averaged, Mw: weight averaged) and polydispersity.

Supplementary Figure S7

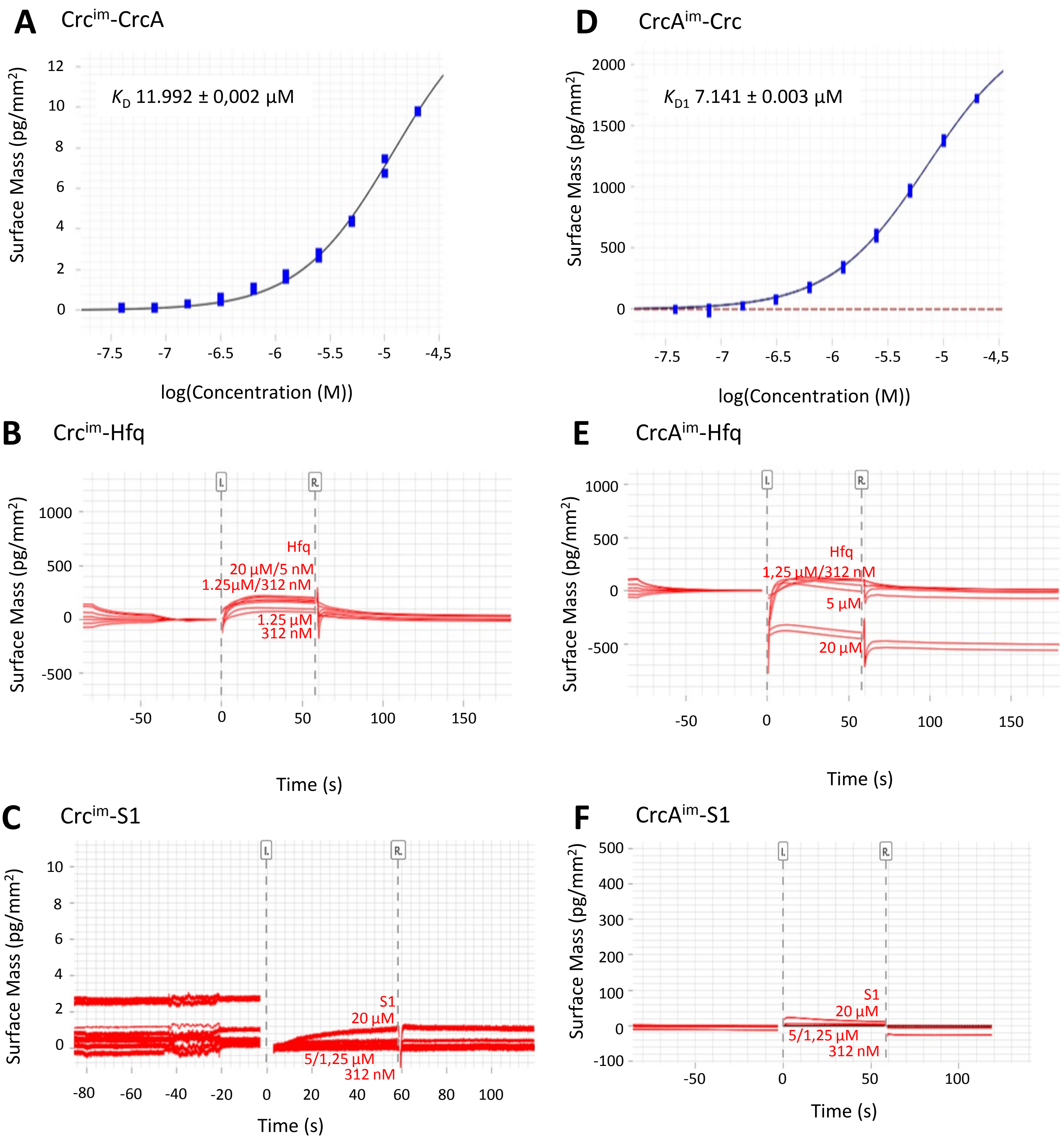

Supplementary Figure S7. Equilibrium curves of the binding kinetics for (**A**) immobilized Crc with CrcA and (**D**) immobilized CrcA with Crc. GCI-sensorgrams of negative controls showing no binding of the immobilized Crc protein (**B**, **C**) and the immobilized CrcA protein (**E**, **F**) with Hfq and ribosomal protein S1, respectively.

3. Supplementary References

Cox, J., and Mann, M. (2008). MaxQuant enables high peptide identification rates, individualized p.p.b.-range mass accuracies and proteome-wide protein quantification. *Nat Biotechnol* 26(12)**,** 1367-1372. doi: 10.1038/nbt.1511.

Fürste, J.P., Pansegrau, W., Frank, R., Blocker, H., Scholz, P., Bagdasarian, M., et al. (1986). Molecular cloning of the plasmid RP4 primase region in a multi-host-range *tac*P expression vector. *Gene* 48(1)**,** 119-131. doi: 10.1016/0378-1119(86)90358-6.

Hmelo, L.R., Borlee, B.R., Almblad, H., Love, M.E., Randall, T.E., Tseng, B.S., et al. (2015). Precision-engineering the *Pseudomonas aeruginosa* genome with two-step allelic exchange. *Nat Protoc* 10(11)**,** 1820-1841. doi: 10.1038/nprot.2015.115.

Holloway, B.W., Krishnapillai, V., and Morgan, A.F. (1979). Chromosomal genetics of *Pseudomonas*. *Microbiol Rev* 43(1)**,** 73-102.

Lüttmann, D., Göpel, Y., and Görke, B. (2012). The phosphotransferase protein EIIA(Ntr) modulates the phosphate starvation response through interaction with histidine kinase PhoR in *Escherichia coli*. *Mol Microbiol* 86(1)**,** 96-110. doi: 10.1111/j.1365-2958.2012.08176.x.

Pettersen, E.F., Goddard, T.D., Huang, C.C., Couch, G.S., Greenblatt, D.M., Meng, E.C., et. al. (2004) UCSF Chimera--a visualization system for exploratory research and analysis. *J Comput Chem.* 25(13), 1605-1612. doi: 10.1002/jcc.20084

Rappsilber, J., Mann, M., and Ishihama, Y. (2007). Protocol for micro-purification, enrichment, pre-fractionation and storage of peptides for proteomics using StageTips. *Nat Protoc* 2(8)**,** 1896-1906. doi: 10.1038/nprot.2007.261.

Rietsch, A., Vallet-Gely, I., Dove, S.L., and Mekalanos, J.J. (2005). ExsE, a secreted regulator of type III secretion genes in *Pseudomonas aeruginosa*. *Proc Natl Acad Sci U S A* 102(22)**,** 8006-8011. doi: 10.1073/pnas.0503005102.

Schnider-Keel, U., Seematter, A., Maurhofer, M., Blumer, C., Duffy, B., Gigot-Bonnefoy, C., et al. (2000). Autoinduction of 2,4-diacetylphloroglucinol biosynthesis in the biocontrol agent *Pseudomonas fluorescens* CHA0 and repression by the bacterial metabolites salicylate and pyoluteorin. *J Bacteriol* 182(5)**,** 1215-1225. doi: 10.1128/jb.182.5.1215-1225.2000.

Simon, R., O'Connell, M., Labes, M., and Puhler, A. (1986). Plasmid vectors for the genetic analysis and manipulation of rhizobia and other gram-negative bacteria. *Methods Enzymol* 118**,** 640-659. doi: 10.1016/0076-6879(86)18106-7.

Zhang, Y.F., Han, K., Chandler, C.E., Tjaden, B., Ernst, R.K., and Lory, S. (2017). Probing the sRNA regulatory landscape of *P. aeruginosa*: post-transcriptional control of determinants of pathogenicity and antibiotic susceptibility. *Mol Microbiol* 106(6)**,** 919-937. doi: 10.1111/mmi.13857.
